# Seneca Valley virus circumvents Gasdermin A-mediated inflammation by targeting the pore-formation domain for cleavage

**DOI:** 10.1128/mbio.01680-24

**Published:** 2024-08-29

**Authors:** Hongyan Yin, Zhenchao Zhao, Ya Yan, Ye Yuan, Weiyu Qu, Haiwei Wang, Cheng Zhu, Pingwei Li, Xin Li

**Affiliations:** 1 National Key Laboratory of Veterinary Public Health and Safety, College of Veterinary Medicine, China Agricultural University, Beijing, China; 2 Key Laboratory of Animal Epidemiology of the Ministry of Agriculture and Rural Affairs, College of Veterinary Medicine, China Agricultural University, Beijing, China; 3 State Key Laboratory of Animal Disease Control, Harbin Veterinary Research Institute, Chinese Academy of Agricultural Sciences, Harbin, China; 4 Tianjin Key Laboratory of Function and Application of Biological Macromolecular Structures, School of Life Sciences, Tianjin University, Tianjin, China; 5 Department of Biochemistry and Biophysics, Texas A&M University, College Station, Texas, USA; Icahn School of Medicine at Mount Sinai, New York, New York, USA; University of California Irvine, Irvine, California, USA

**Keywords:** Gasdermin A, Seneca Valley Virus, 3C protease, cleavage, pyroptosis

## Abstract

Members of the gasdermin (GSDM) family are critical for inducing programmable pyroptosis by forming pores on the cell membrane. GSDMB, GSDMC, GSDMD, and GSDME are activated by caspases or granzyme, leading to the release of their autoinhibitory domains. The protease SpeB from group A *Streptococcus* has been shown to cleave and activate GSDMA-mediated pyroptosis. Meanwhile, African Swine Fever Virus infection regulates pyroptosis by cleaving porcine GSDMA (pGSDMA) via active caspase-3 and caspase-4. However, it is not known whether virus-encoded proteases also target GSDMA. Here, we show that residues 1–252 of pGSDMA (pGSDMA_1–252_) is the pore-forming fragment that induces lytic cell death and pyroptosis. Interestingly, Seneca Valley Virus (SVV) infection induces the cleavage of both pGSDMA and human GSDMA and suppresses GSDMA-mediated cell death. Mechanistically, SVV protease 3C cleaves pGSDMA between Q187 and G188 to generate a shorter fragment, pGSDMA_1–186_, which fails to induce lytic cell death and lactate dehydrogenase release. Furthermore, pGSDMA_1–186_ does not localize to the plasma membrane and does not induce cell death, thereby promoting viral replication by suppressing host immune responses. These studies reveal a sophisticated evolutionary adaptation of SVV to bypass GSDMA-mediated pyroptosis, allowing it to overcome host inflammatory defenses.

**IMPORTANCE:**

Gasdermin A (GSDMA) remains a protein shrouded in mystery, particularly regarding its regulation by virus-encoded proteases. Previous studies have identified human GSDMA (hGSDMA) as a sensor and substrate of the SpeB from group A *Streptococcus*, which initiates pyroptosis. However, it is not clear if viral proteases also cleave GSDMA. In this study, we show that a fragment of porcine GSDMA (pGSDMA) containing the first 252 residues constitutes the pore-forming domain responsible for inducing lytic cell death and pyroptosis. Interestingly, picornavirus Seneca Valley Virus (SVV) protease 3C cleaves both pGSDMA and hGSDMA, generating a shorter fragment that fails to associate with the plasma membrane and does not induce pyroptosis. This cleavage by SVV 3C suppresses GSDMA-mediated lactate dehydrogenase release, bactericidal activity, and lytic cell death. This study reveals how SVV subverts host inflammatory defense by disrupting GSDMA-induced pyroptosis, thereby advancing our understanding of antiviral immunity and opening avenues for treating GSDMA-associated autoimmune diseases.

## INTRODUCTION

Gasdermins (GSDMs) are integral to innate immunity, playing a critical role in the defense against intracellular pathogens (1–3). Humans express six GSDM family members: GSDMA, GSDMB, GSDMC, GSDMD, GSDME, and pejvakin (4). GSDMD has been the focus of extensive research, and its activation mechanisms are well-understood (5–7). Under resting conditions, the N-terminal domain (NTD) of GSDMD is inhibited by its C-terminal domain (CTD), a process known as autoinhibition (8). Activation of GSDMD occurs when caspase-1 cleaves the interdomain linker, prompting GSDMD NTD to polymerize and form pores in the plasma membrane, leading to lytic cell death and the release of interleukin-1 beta (IL-1β) (5).

Many picornaviruses have been reported to induce pyroptosis by cleaving different GSDM family proteins. Enterovirus A71 (EV71) and coxsackievirus B3 (CVB3) infections lead to the activation of caspase-1 to induce pyroptosis, which affects viral replication and host responses (9–11). EV71 infection also induces GSDME-mediated pyroptosis by activating caspase-3 (12). Foot-and-mouth disease virus 3C cleaves porcine GSDME at the Q271-G272 junction, adjacent to the cleavage site (D268-A269) of porcine caspase-3, to induce pyroptosis (13). Seneca Valley Virus (SVV) 3C cleaves porcine GSDMD at Q193 and Q277 to induce pyroptosis in SK6 cells, and Q277 is close to the caspase-1 cleavage site of porcine GSDMD (14). Meanwhile, viruses have developed numerous tactics to counteract GSDM-mediated pyroptosis. For example, EV71 protease 3C directly cleaves GSDMD into a shorter N-terminal fragment, GSDMD_1–193_, instead of the pore-forming fragment, GSDMD_1–275_. This fragment reduces the ability of GSDMD to induce pyroptosis, thereby enhancing EV71 replication (15). Furthermore, the nucleocapsid of severe acute respiratory syndrome coronavirus 2 (SARS-CoV-2) specifically interacts with the GSDMD linker region, thus inhibiting caspase-1-mediated GSDMD processing in human monocytes (16). African swine fever virus (ASFV) protease pS273R cleaves porcine GSDMD to generate GSDMD_1–107_, which mitigates the inflammatory response more effectively than GSDMD_1–279_ (17). Similarly, porcine epidemic diarrhea virus (PEDV) produces a truncated GSDMD_1–193_, instead of the fragment GSDMD_1–279_, modulating pyroptosis (18). Collectively, these mechanisms highlight viral strategies to broadly suppress host pyroptosis, culminating in the controlled release of mature IL-1β (19).

Substrate specificity allows distinct GSDM proteins to be selectively cleaved by particular caspases. For example, human GSDMD can be cleaved by caspase-1, caspase-4, caspase-5, and caspase-11 (5, 7, 20), while mouse GSDMD is a substrate of caspase-11 (21). GSDME can be cleaved by caspase-3 (22), and GSDMB responds to granzyme A (23). GSDMC is a substrate for caspase-8 (24), and non-mammal GSDMA is known to be cleaved and activated by caspase-1 (25). A cysteine protease from group A *Streptococcus*, SpeB, is the only known pathogen protease that cleaves and activates human GSDMA (hGSDMA), triggering pyroptosis (26, 27). Recently, Li et al. reported that ASFV infection induces pyroptosis by cleaving porcine GSDMA (pGSDMA) via active porcine caspase-3 (pcaspase-3) and pcaspase-4 (28). GSDMA is highly expressed in the skin epithelial keratinocytes (26, 27), yet the capability of viral protease to cleave GSDMA or the mechanisms behind non-human mammalian GSDMA activation remain largely unexplored.

In 2016, the Shao group reported that hGSDMA_1–251_ domain can induce mammalian cell pyroptosis and exhibit cytotoxicity in bacteria (1). In this study, we demonstrate that pGSDMA contains an active pore-forming fragment, pGSDMA_1–252_, which inserts into the plasma membrane and oligomerizes, leading to pore formation and lytic cell death. SVV infection causes the cleavage of both pGSDMA and hGSDMA. The SVV protease 3C specifically cleaves pGSDMA between Q187 and G188 and hGSDMA between Q186 and G187, producing shorter N-terminal (NT) fragments, pGSDMA_1–186_ and hGSDMA_1–185_, respectively. These shorter fragments fail to localize to the plasma membrane and do not promote lactate dehydrogenase (LDH) release, bactericidal activity, and pyroptosis, thereby facilitating SVV replication. These results reveal a novel strategy by which the SVV circumvent GSDMA-mediated inflammatory restriction.

## RESULTS

### The NTD of pGSDMA induces pyroptosis

**Fig 1:**
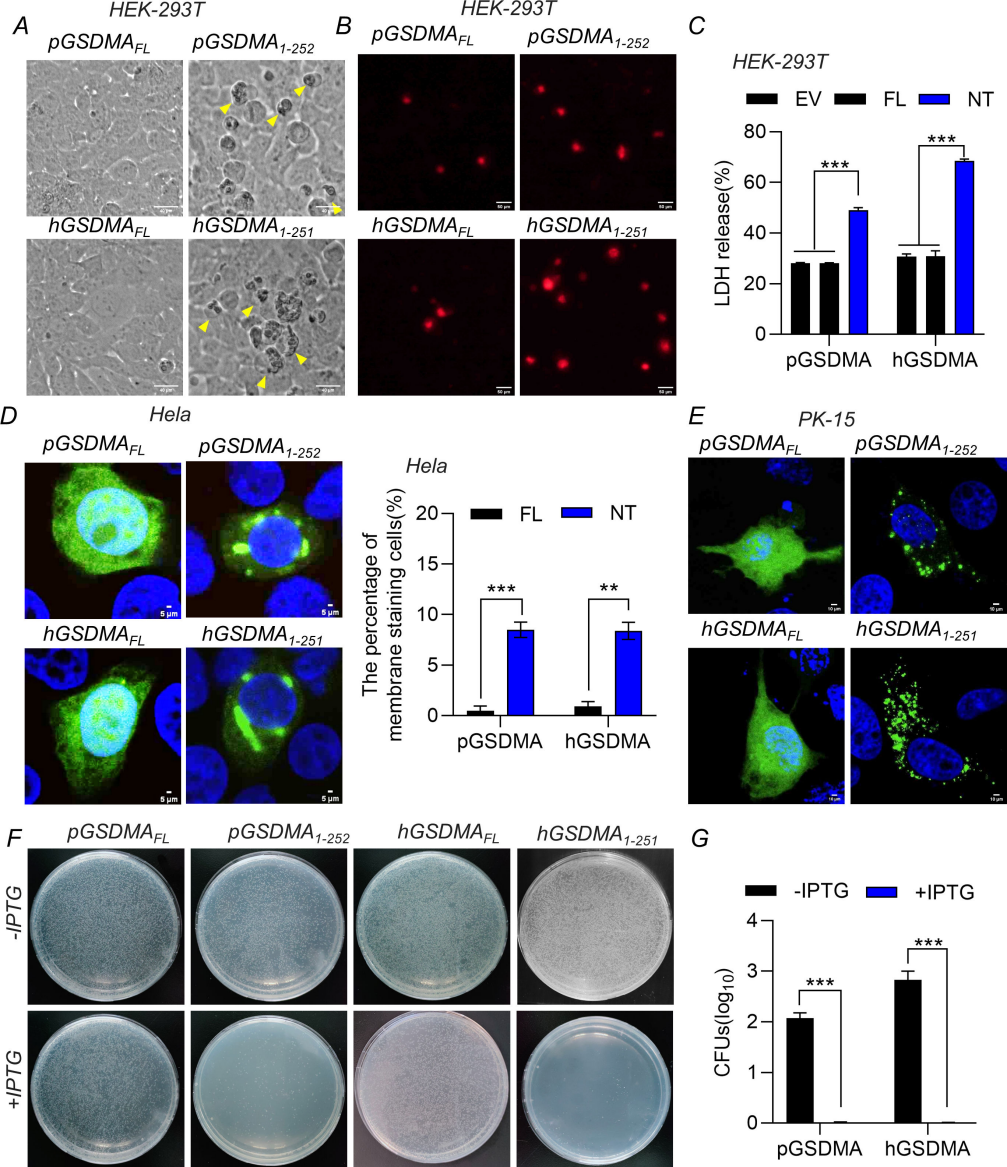
The NTD of pGSDMA induces pyroptosis. (**A–C**) HEK-293T cells were transfected with a plasmid encoding full length or NTD from both pGSDMA and hGSDMA for 24 h. (**A**) Morphological change characteristics of pyroptosis were visualized using light microscopy, with arrows highlighting pyroptotic cells. Scale bar, 40 µm. (**B**) The cells were stained with PI and analyzed with fluorescence microscopy. Scale bar, 50 µm. (**C**) The supernatants were collected and analyzed for LDH levels at optical density (OD) at 490 nm. (**D and E**) Immunofluorescence analysis of the localization of pGSDMA or hGSDMA NTD in Hela cells (**D**) and PK-15 cells (**E**) transfected with a plasmid encoding GSDMA full length or NTD from pig and human for 24 h, followed by fixation and subsequently staining with rabbit monoclonal-specific Abs for V5 (green) and specific secondary antibodies. Nuclei were stained with 4',6-diamidino-2-phenylindole (DAPI, blue). The fluorescent signals were observed with confocal immunofluorescence microscopy. Cumulative data of membrane staining cells in Hela cells of panel D were in the right. Scale bar, 50 µm. (**F and G**) *E. coli* BL21 was transfected with a pET-28a plasmid expressing GSDMA full length or NTD. The transformants were cultured inLuria-Bertani (LB) medium containing 50 μg/mL kanamycin as above to OD^_600_^ 0.6. Cells were diluted and grown on kan^+^ LB agar plates with or without 0.4 mM isopropyl β-D-1-thiogalactopyranoside (IPTG). After incubation at 37°C overnight, colony-forming unit (CFU) on the plates was counted and statistically calculated. Cumulative data were shown in the right (**G**). Data are represented as means ± SD from three biological replicates. **P < 0.01, ***P < 0.001, Student’s *t*-test.

Previous studies demonstrated that the NTD (1–251 aa) of hGSDMA triggers pyroptosis in human HEK-293T cells (human embryonic kidney cells) (1); we hypothesized that the NTD of pGSDMA likely also disrupts cellular membranes to induce pyroptosis. Amino acid sequence alignment of GSDMA from pig, human, and other species reveals a high degree of conservation, particularly within the first 252 amino acids of pGSDMA (Fig. S1A). To explore the pyroptotic potential of pGSDMA NTD, we transfected HEK-293T cells with plasmids encoding full length and the NTD of pGSDMA and hGSDMA for 24 h. Cells expressing GSDMA NTD exhibited characteristic morphological changes consistent with pyroptosis, including rounding, swelling, and a “fried-egg” appearance due to the protruding nucleus (Fig. 1A). Consistent with these morphological features, the percentage of propidium iodide^+^ (PI) cells and the release of LDH were dramatically increased in HEK-293T cells transfected with pGSDMA NTD plasmid (Fig. 1B and C). Like other GSDM family proteins, the NTDs are known for their membrane-disrupting abilities, affinity for cardiolipin, and bactericidal properties (1). We investigated the subcellular localization of both full-length and the NTD of pGSDMA and hGSDMA. The truncated forms, pGSDMA_1–252_ and hGSDMA_1–251_, predominantly localized near the plasma membrane, whereas full-length hGSDMA and pGSDMA were diffusely distributed within Hela and PK-15 cells (porcine kidney-15) (Fig. 1D and E). Furthermore, to assess the bactericidal activity of pGSDMA NTD, *Escherichia coli* BL21 cells were transformed with plasmids encoding full-length or the NTD of GSDMA. Upon induction, the bacterial count significantly declined with pGSDMA_1–252_ and hGSDMA_1–251_ expressions but not with the expression of pGSDMA_FL_ and hGSDMA_FL_ (Fig. 1F and G). Collectively, these studies confirmed the inherent cytotoxicity of the pGSDMA NTD, which prompts pyroptosis upon overexpression in mammalian cells.

### SVV infection induces the cleavage of pGSDMA and hGSDMA

**Fig 2:**
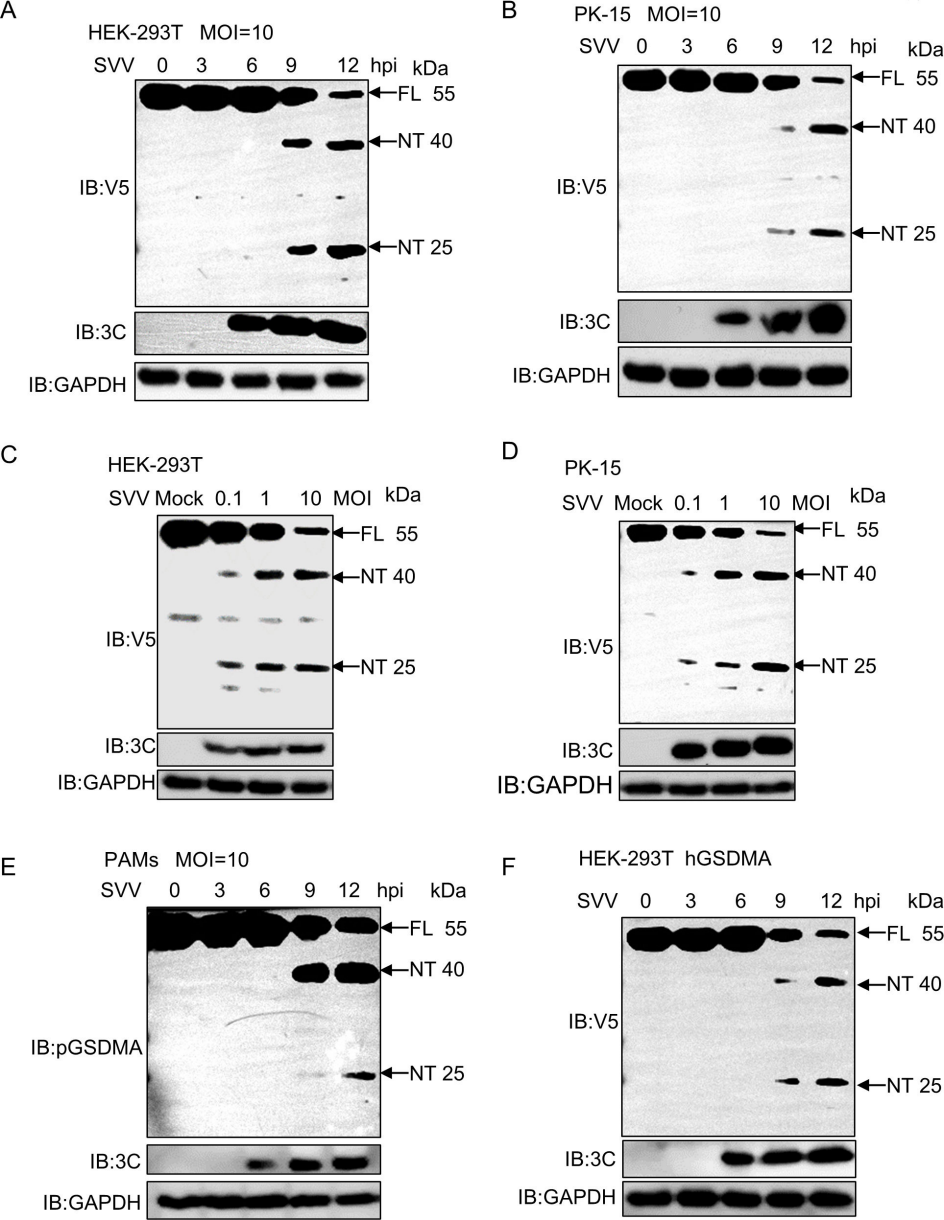
SVV infection induces the cleavage of pGSDMA and hGSDMA. (**A and B**) Immunoblotting analysis of the protein expression of pGSDMA in HEK-293T cells (**A**) and PK-15 cells (**B**) transfected with a plasmid encoding full-length pGSDMA for 24 h, followed by SVV infection (MOI = 10) for the indicated time points. (**C and D**) Immunoblotting analysis of the protein expression of pGSDMA in HEK-293T cells (**C**) and PK-15 cells (**D**) transfected with a plasmid encoding full-length pGSDMA for 24 h, followed by SVV infection with different doses (MOI = 0.1, 1, 10) for 24 h. (**E**) Immunoblotting analysis of the endogenous protein expression of pGSDMA in PAMs infected with SVV (MOI = 10) for indicated time points. (**F**) Immunoblotting analysis of hGSDMA expression in HEK-293T cells transfected with a plasmid encoding full-length hGSDMA for 24 h, followed by SVV infection with (MOI = 10) for the indicated time points.

To investigate the effect of SVV infection on pGSDMA expression, HEK-293T cells and PK-15 cells overexpressing pGSDMA were infected with SVV at the indicated time points. Immunoblotting showed that the protein level of pGSDMA was reduced, as evidenced by two distinct cleavage bands with molecular mass (~40 kDa and 25 kDa) at 9 and 12 h post SVV infection (Fig. 2A and B). Similarly, pGSDMA protein level was reduced upon SVV infection at different multiplicity of infection (MOI) (0.1, 1, 10) in HEK-293T cells and PK-15 cells (Fig. 2C and D). To detect the endogenous expression of pGSDMA, we analyzed its gene expression across various tissues and cells and observed a predominant expression of pGSDMA in the skin and porcine alveolar macrophages (PAMs) (Fig. S1B). Recombinant pGSDMA was expressed in *E. coli* and purified by gel filtration and ion exchange chromatography (Fig. S2A and B). The purified protein was used to generate polyclonal antibody (pAb) against pGSDMA in mice (Fig. S2C). To provide a physiological context, we isolated the primary PAMs by alveolar lavage fluid and the PAMs were infected with SVV at 0, 3, 6, 9, and 12 h. The results confirmed that SVV infection caused the cleavage of pGSDMA into two fragments on endogenous level (Fig. 2E). Moreover, to confirm whether SVV infection similarly affects hGSDMA expression, HEK-293T cells expressing hGSDMA were infected with SVV at indicated time points. Consistently, the results showed that SVV infection also caused the cleavage of hGSDMA into two NT (~40 kDa and ~25 kDa) fragments (Fig. 2F). In summary, SVV infection of different type of cells reduces both pGSDMA and hGSDMA levels through proteolytic cleavage.

### SVV 3C protease cleaves pGSDMA between Q187 and G188

**Fig 3:**
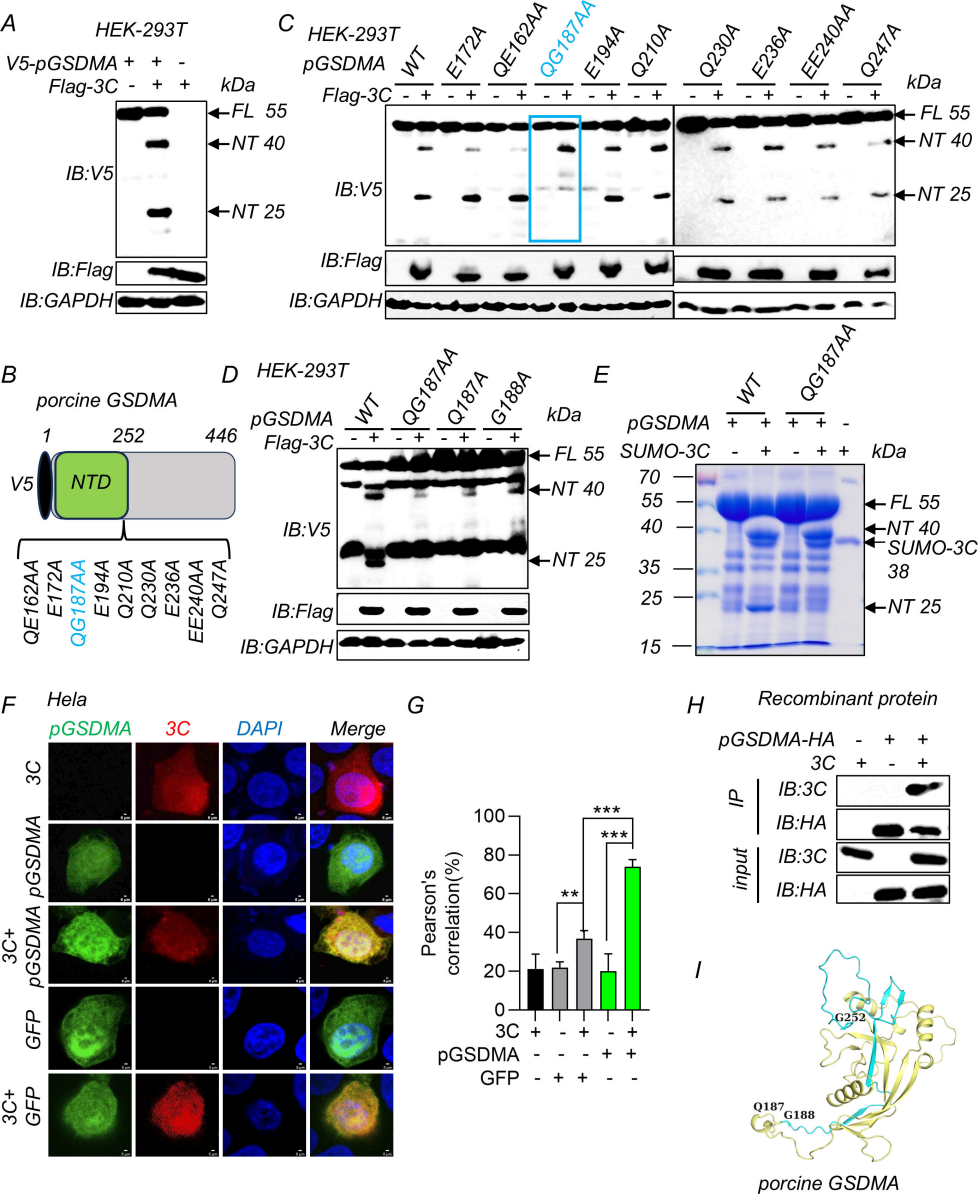
SVV 3C protease cleaves pGSDMA between Q187 and G188. (**A**) Immunoblotting analysis of the cleavage pGSDMA band in HEK-293T cells co-transfected with plasmids encoding SVV 3C and full-length pGSDMA for 24 h. (**B**) Schematic representation of V5-pGSDMA and its mutants. (**C**) Immunoblotting analysis of the cleavage band in HEK-293T cells co-transfected with plasmids encoding SVV 3C and pGSDMA mutants as indicated for 24 h. (**D**) Immunoblotting analysis of the cleavage band in HEK-293T cells co-transfected with plasmids encoding SVV 3C and wild-type (WT) or mutants pGSDMA (QG187AA, Q187A, or G188A) for 24 h. (**E**) Cleavage of pGSDMA and pGSDMA-QG187AA recombinant protein by SVV SUMO-3C *in vitro*. (**F and G**) The confocal microscopy analysis of colocalization between pGSDMA and SVV 3C in Hela cells co-transfected with plasmids encoding SVV 3C and full-length pGSDMA or green fluorescent protein (GFP) for 24 h, and followed labeling with Flag-3C- and V5-pGSDMA-specific primary antibodies and secondary antibodies (red and green). GFP as negative control. Cell nuclei were stained with 4',6-diamidino-2-phenylindole (DAPI, blue). The fluorescent signals were observed with confocal immunofluorescence microscopy. Pearson correlation (%) analysis was shown in right (**G**). (**H**) Coimmunoprecipitation (Co-IP) assay for assessment of the interactions between pGSDMA and 3C recombinant proteins *in vitro*, which were immunoprecipitated against rabbit anti-hemagglutinin (HA) monoclonal antibody. (**I**) The pGSDMA NTD model with SVV 3C cleavage sites by AlphaFold website. Blue and orange cartoon, active NTD domain; blue cartoon, inactive fragment. Data are represented as means ± SD from three biological replicates. ***P < 0.001, Student’s *t*-test.

To investigate the specificity of SVV 3C protease for pGSDMA, we co-transfected HEK-293T cells with plasmids encoding Flag-SVV 3C and V5-pGSDMA. Subsequent immunoblotting revealed two distinct pGSDMA bands, approximately 40 kDa and 25 kDa in sizes (Fig. 3A). To test whether SVV 3C directly cleaves pGSDMA, we incubated purified, full-length pGSDMA with recombinant SVV 3C protease and observed that SVV 3C cleaved recombinant pGSDMA in a dose-dependent manner, resulting in an NT (~40 kDa) and a CT (~15 kDa) fragment (Fig. S3A and B). Mass spectrometry analysis identified the initial peptide of the cleavage product beginning from K379, near the ~40 kDa marker (Fig. S3C). Next, a variety of pGSDMA deletion mutants were engineered to investigate the cleavage mechanism. Despite these alterations, co-transfection with SVV 3C consistently yielded the 40 kDa NT fragment, indicating that none of the mutations disrupted this specific cleavage event (Fig. S3D). Based on the results from the mutagenesis studies, we hypothesized that this K379 (~40 kDa) fragment is an intermediate product and could be further cleaved either by endogenous enzyme into pGSDMA_1–252_ active fragment or by SVV 3C protease into the 25 kDa fragment. Previously, in our recombinant protein assays, we could only detect the pGSDMA NT (~40 kDa) but not the smaller NT fragment (~25 kDa). It was likely that SVV 3C (~23 kDa) and pGSDMA NT (~25 kDa) overlapped in SDS-PAGE due to these similar sizes, obscuring the ~25 kDa band (Fig. S3A and B). To clarify this, we purified a larger SVV SUMO-3C (~38 kDa) protein to perform the cleavage assay (Fig. S4A). The results showed SVV SUMO-3C cleaved full-length pGSDMA protein, producing both NT (~40 kDa) and NT (~25 kDa) fragments (Fig. S4B, right). We refolded and purified the truncated SUMO-pGSDMA-p40 (1–379 aa, ~65 kDa) recombinant protein to determine whether 3C could cleave this truncated pGSDMA protein (Fig. S4C). Furthermore, SVV 3C cleaved recombinant SUMO-pGSDMA-p40 to yield a ~40 kDa SUMO-N-terminal (SUMO tag 15 kDa plus NT 25 kDa) (Fig. S4D). Taken together, these results indicate that SVV 3C cleaves fully folded protein and its truncation, resulting in a cleavage product of ~25 kDa.

Previous studies suggest picornavirus 3C protease preferentially recognizes glutamine-glycine (Q-G) or glutamic acid-glutamine (E-Q) site (29). Based on the potential biological functions of cleaved NT fragment (~25 kDa), we constructed a series of pGSDMA mutants by replacing the glutamine and glutamic residues at the potential cleavage site with alanine residues (Fig. 3B). SVV 3C and pGSDMA mutant plasmids were co-transfected in HEK-293T cells. Immunoblotting analysis showed that the Q187A/G188A mutation of pGSDMA eliminated the 25 kDa NT cleavage fragment, leaving the larger fragment (~40 kDa) unaffected, which confirmed that 25 kDa NT is the cleavage product of SVV 3C protease (Fig. 3C and D). Because the cleavage sites of pGSDMA by SVV 3C are located between Q187 and G188, we purified recombinant mutant pGSDMA-QG187AA protein to perform *in vitro* cleavage assay (Fig. S5A and B). SDS-PAGE analysis showed that SVV SUMO-3C protease cleaved recombinant full-length pGSDMA into two distinct NT fragments, approximately 40 kDa and 25 kDa in size. In contrast, cleavage of pGSDMA-QG187AA yielded only the ~40 kDa NT fragment, but not the ~25 kDa NT fragment (Fig. 3E). These results confirmed that SVV 3C cleaved pGSDMA between Q187 and G188. To explore whether SVV 3C colocalizes with pGSDMA, immunofluorescence analysis demonstrated that SVV 3C colocalized with pGSDMA in both the cytoplasm and nucleus when co-expressing SVV 3C and pGSDMA (Fig. 3F and G). Consistently, Pearson’s analysis suggested a significant difference between pGSDMA with 3C group and GFP with 3C group (Fig. 3G), suggesting the colocalization was specific. In addition, we conducted coimmunoprecipitation assay using full-length pGSDMA and WT 3C recombinant proteins and confirmed the direct interaction between SVV 3C and pGSDMA in a cell-free system (Fig. 3H). In addition, we have generated a pGSDMA structural model by AlphaFold based on the mouse GSDMA3 crystal structure (PDB: 5B5R). As shown in Fig. 3I, the orange plus blue cartoon represents the NTD (1–252 aa) with pore-forming activity and the orange cartoon represents the inactive fragment (1–186 aa), which was generated by SVV 3C cleavage and inhibits lytic cell pyroptosis (Fig. 3I). To determine whether SVV 3C protease activity was essential for the cleavage of pGSDMA, SVV 3C WT and its catalytic mutant proteins were co-incubated with recombinant pGSDMA. No obvious cleavage was observed with the SVV 3C mutants, consistent with the co-transfection data in HEK-293T cells (Fig. S5C and D). Together, these results suggest that SVV 3C protease cleaves pGSDMA and its catalytic activity is required for pGSDMA cleavage.

### SVV 3C protease cleaves hGSDMA between Q186 and G187

**Fig 4:**
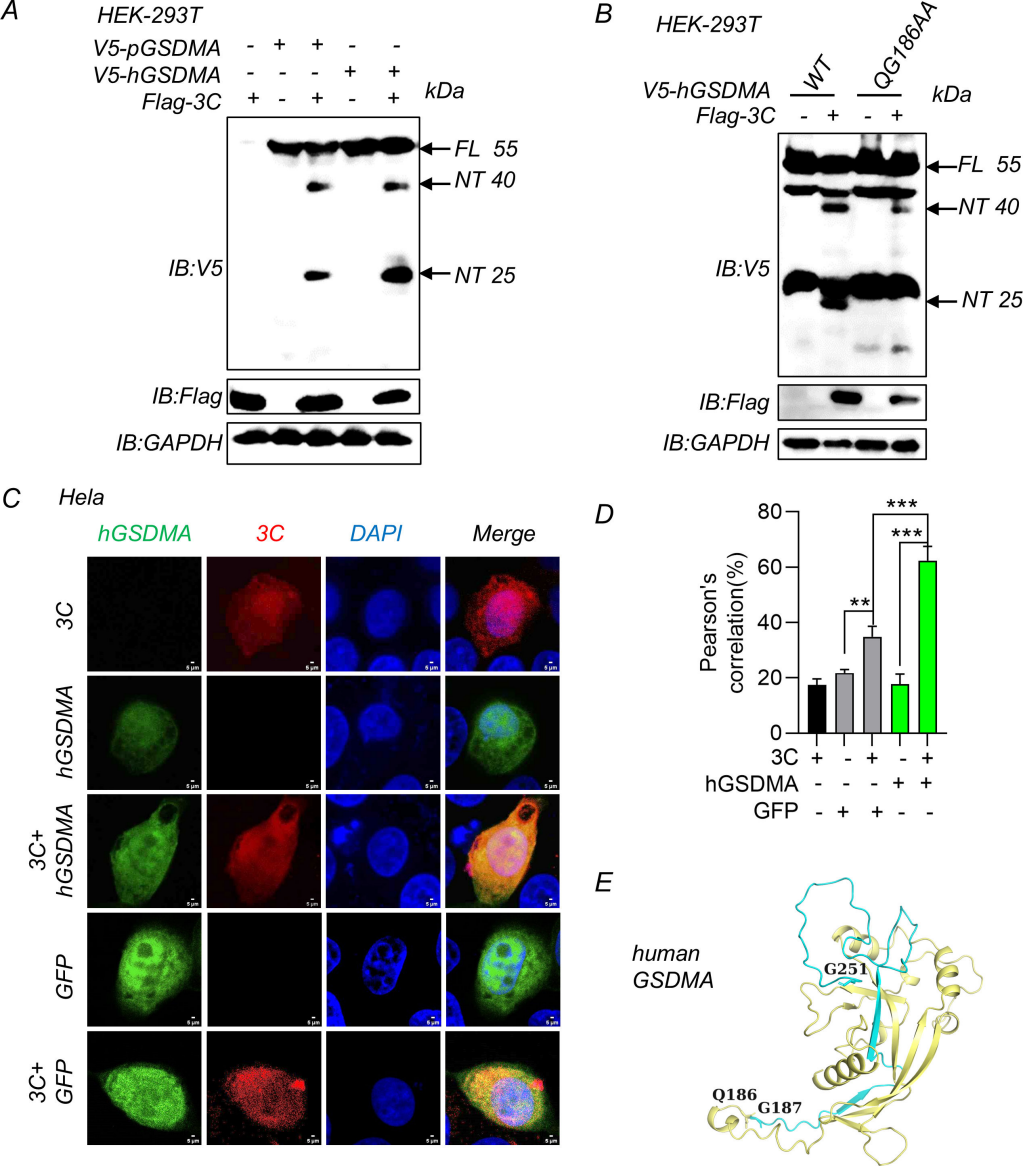
SVV 3C protease cleaves hGSDMA between Q186 and G187. (**A**) Immunoblotting analysis of the hGSDMA cleavage band in HEK-293T cells co-transfected with plasmids encoding SVV 3C and full-length pGSDMA or hGSDMA for 24 h. (**B**) Immunoblotting analysis of the hGSDMA cleavage in HEK-293T cells were co-transfected with plasmids encoding SVV 3C and hGSDMA mutants (WT or QG186AA) for 24 h. (**C and D**) The confocal microscopy analysis of colocalization between hGSDMA and SVV 3C in Hela cells co-transfected with plasmids encoding SVV 3C and full-length hGSDMA or green fluorescent protein (GFP) for 24 h, and then labeled with Flag-3C- and V5-hGSDMA-specific primary antibodies and secondary antibodies (red and green). GFP as a negative control. Cell nuclei were stained with 4',6-diamidino-2-phenylindole (DAPI, blue). The fluorescent signals were observed with confocal immunofluorescence microscopy. Pearson correlation (%) analysis was shown in right (**D**). (**E**) The hGSDMA NTD model with SVV 3C cleavage sites by AlphaFold website. Blue and orange cartoon, active NTD domain; blue cartoon, inactive fragment. Data are representative of those from three independent experiments. Data are represented as means ± SD from three biological replicates. ***P < 0.001, Student’s *t*-test.

Given the strong sequence similarity between pGSDMA and hGSDMA and the conserved cleavage site sequence, we hypothesized that SVV 3C protease likely also cleaves hGSDMA. Immunoblotting analysis showed that hGSDMA was cleaved by SVV 3C into two fragments (~40 kDa and 25 kDa) when SVV 3C was co-expressed with hGSDMA in HEK-293T cells (Fig. 4A). Consistent with these results, when we co-expressed SVV 3C with hGSDMA QG186AA mutant, the NT (~25 kDa) cleavage fragment disappeared (Fig. 4B). Immunofluorescence analysis showed that 3C colocalized with hGSDMA when co-expressing SVV 3C and hGSDMA in Hela cells (Fig. 4C and D). Consistently, Pearson’s analysis suggested a significant difference between hGSDMA with 3C group and GFP with 3C group (Fig. 4D). Similarly, we generated an hGSDMA structural model by AlphaFold based on the mouse GSDMA3 crystal structure (PDB: 5B5R). As shown in Fig. 4E, the orange plus blue cartoon represented the NTD (1–251 aa) with pore-forming activity, while the orange cartoon represented the inactive fragment (1–185 aa). The shorter fragment of hGSDMA (1–185 aa) produced by SVV 3C cleavage interferes with the functional fragment (1–251 aa) in suppressing pyroptosis (Fig. 4E). Thus, these results indicate that SVV 3C cleaves both porcine and human GSDMA.

### pGSDMA_1–186_ fails to induce pyroptosis

**Fig 5:**
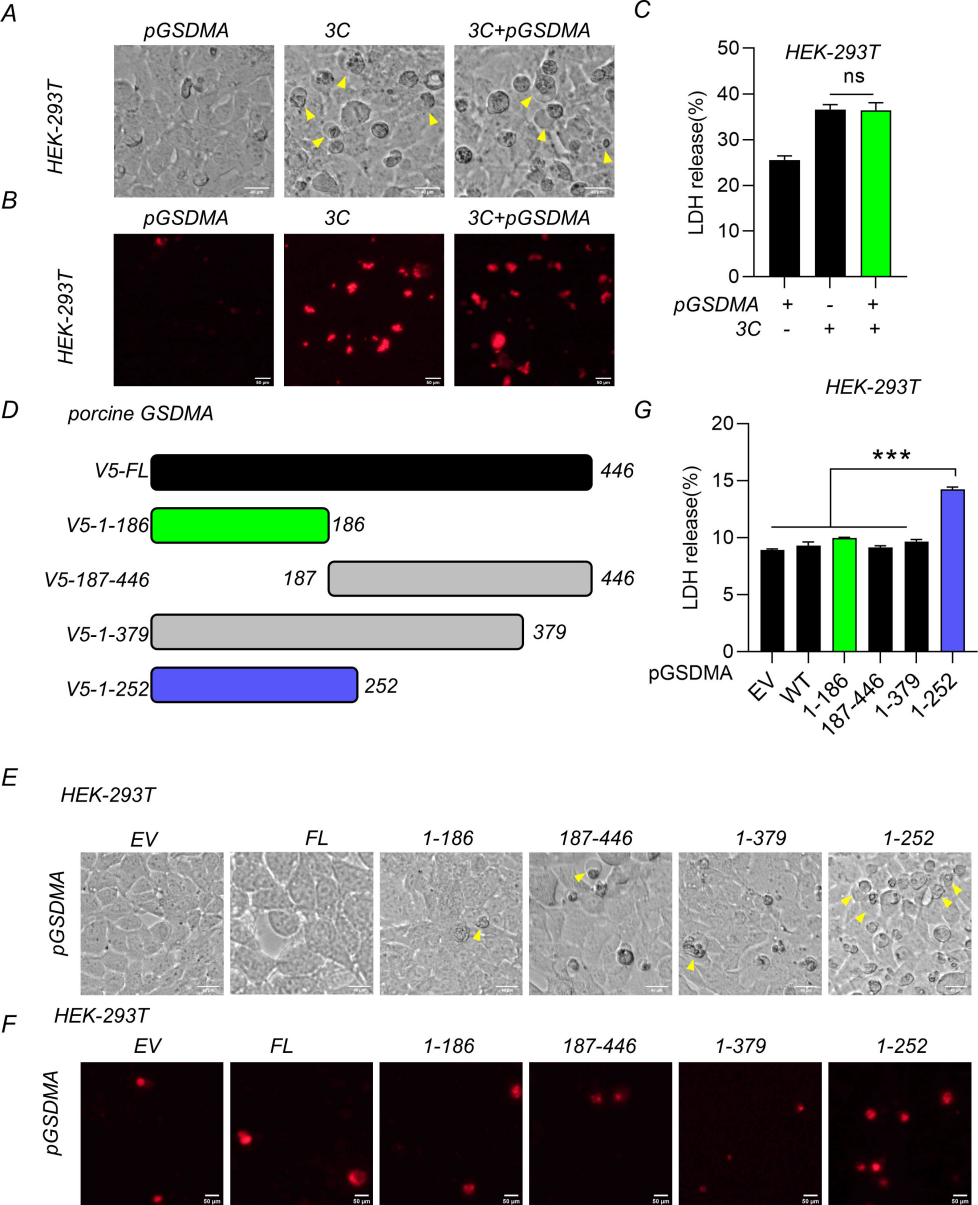
pGSDMA_1–186_ fails to induce pyroptosis. (**A–C**) HEK-293T cells were transfected with plasmids encoding SVV 3C and full-length pGSDMA for 24 h. (**A**) Morphological change characteristics of pyroptosis were visualized using light microscopy, with arrows highlighting pyroptotic cells. Scale bar, 40 µm. (**B**) The cells were stained with PI for 30 min at 37°C and analyzed with fluorescence microscopy. Scale bar, 50 µm. (**C**) The supernatants were collected and analyzed for LDH levels at OD = 490 nm. (**D**) Schematic representation of V5-pGSDMA and its truncations. (**E–G**) HEK-293T cells were transfected with a plasmid encoding different truncations (full length, 1–186, 187–446,1-379, or 1–252 aa) of pGSDMA for 24 h. (**E**) Morphological change characteristics of pyroptosis were visualized using light microscopy, with arrows highlighting pyroptotic cells. Scale bar, 40 µm. (**F**) The cells were stained with PI and analyzed with fluorescence microscopy. Scale bar, 50 µm. (**G**) The supernatants were collected and analyzed for LDH levels at OD = 490 nm. Data are represented as means ± SD from three biological replicates. ns, no significance, ***P < 0.001, Student’s *t*-test.

SVV 3C was known to cause significant cytopathic effects and lytic cell death (14). To investigate the roles of pGSDMA cleavage by SVV 3C in cell death, SVV 3C and pGSDMA plasmids were co-transfected in HEK-293T cells. Firstly, we observed that pGSDMA cleavage by SVV 3C did not significantly influence the number of cells displaying characteristic “bubble-like” features of pyroptosis compared with the expression of SVV 3C alone (Fig. 5A). This observation was corroborated by PI staining and LDH release assays (Fig. 5B and C). Similarly, the cleavage fragment of hGSDMA, when co-expressed with SVV 3C in HEK-293T cells, also failed to induce pyroptosis (Fig. S6A through C). These findings indicate that the NT fragments of both pGSDMA and hGSDMA generated by SVV 3C cleavage are not capable of triggering pyroptosis.

To determine the effect of SVV 3C-mediated cleavage of pGSDMA on pyroptosis, we constructed different truncated pGSDMA plasmids (Fig. 5D). Notably, the three cleavage fragments including pGSDMA_1–186_, pGSDMA_187–446_, and pGSDMA_1–379_ were unable to induce pyroptosis as indicated by morphological feature change, PI staining and LDH release analysis (Fig. 5E through G). Similarly, hGSDMA_1–185_ did not induce cell death based on the observation of typical pyroptosis morphological features and LDH release (Fig. S6D through G). Taken together, these results demonstrated that SVV 3C cleaves both pGSDMA and hGSDMA to produce a shorter fragment that is not capable to induce pyroptosis.

### The pGSDMA_1–186_ fragment fails to localize to membrane and loses its bactericidal activity

**Fig 6:**
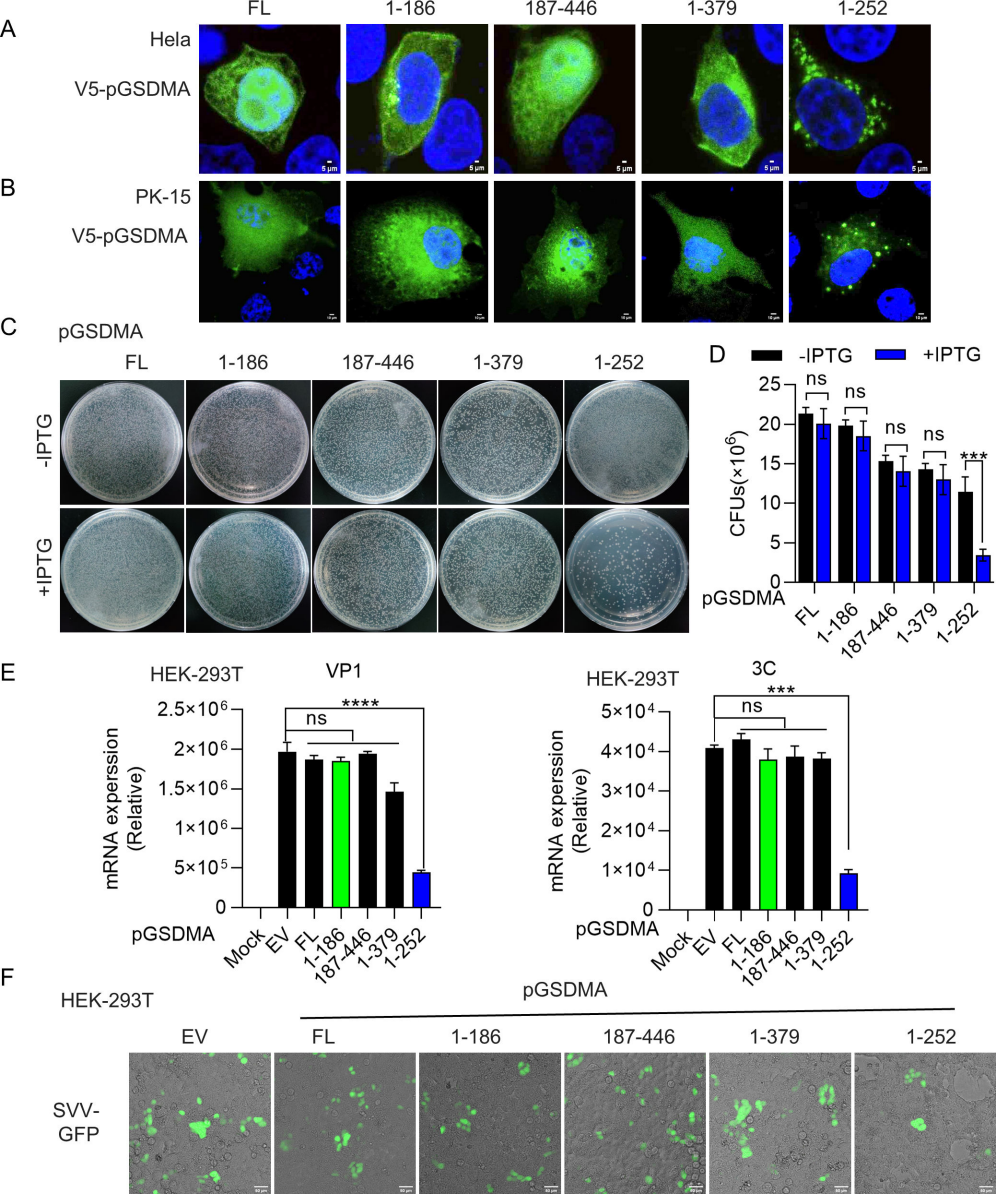
pGSDMA_1–186_ fails to localize to the membrane and loses its bactericidal activity. (**A and B**) Immunofluorescence analysis of the localization of pGSDMA cleaved fragments in Hela cells (**A**) and PK-15 (**B**) cells transfected with a plasmid encoding different truncations (full length, 1–186, 187–446,1-379, or 1–252 aa) of pGSDMA for 24 h, followed by fixation and subsequently staining with rabbit monoclonal-specific Abs for V5 (green). Nuclei were stained with 4',6-diamidino-2-phenylindole (DAPI, blue). The fluorescent signals were observed with confocal immunofluorescence microscopy. (**A**) Scale bar, 5 µm. (**B**) Scale bar, 10 µm. (**C and D**) *E. coli* BL21 was transfected with a pET-28a plasmid expressing different truncations (full length, 1–186, 187–446,1-379, or 1–252 aa) of pGSDMA. The transformants were cultured inluria-bertani (LB) medium containing 50 μg/mL kanamycin as above to OD_600_ 0.6. Cells were diluted and grown on kan^+^ LB agar plates with or without 0.4 mM IPTG. After incubation at 37°C overnight, colony-forming unit (CFU) on the plates was counted and statistically calculated. (**D**) Cumulative data were shown in the right. (**E**) Real time polymerase chain reaction (RT-PCR) analysis of VP1 and 3C mRNA expression of SVV in HEK-293T cells transfected with a plasmid encoding different truncations (full length, 1–186, 187–446,1-379, or 1–252 aa) of pGSDMA for 24 h. (**F**) Fluorescence analysis of GFP-positive cells in HEK-293T cells processed as above, followed by infection with SVV-GFP (MOI = 1). Scale bar, 50 µm. Data are represented as means ± SD from three biological replicates. ns, no significance, ***P < 0.001, ****P < 0.0001, Student’s *t*-test.

Our previous results showed that pGSDMA_1–252_ localized to the plasma membrane and exhibited bactericidal activity. To confirm whether pGSDMA mutants localize on the cell membrane, pGSDMA_1–252_ and pGSDMA truncated plasmids were transfected into the Hela and PK-15 cells. We observed that pGSDMA_1–186_ was diffusely distributed in the cells compared with the positive control, while pGSDMA_1–252_ mainly formed aggregates around the plasma membrane (Fig. 6A and B). The shorter hGSDMA fragments were also unable to localize to the membrane in the cells compared with hGSDMA_1–251_ (Fig. S7A and B). Because the lytic cell death caused GSDMA NTD, the number of *E. coli* cells expressing pGSDMA_1–252_ significantly decreased after isopropyl β-D-1-thiogalactopyranoside (IPTG) induction, whereas no cytotoxicity was observed for pGSDMA_1–186_ and other shorter fragments (Fig. 6C and D). Similar results were observed with hGSDMA (Fig. S7C and D). Collectively, the shorter cleavage fragments of both pGSDMA and hGSDMA generated by SVV 3C protease fail to localize to the membrane and do not have bactericidal activity.

**Fig 7:**
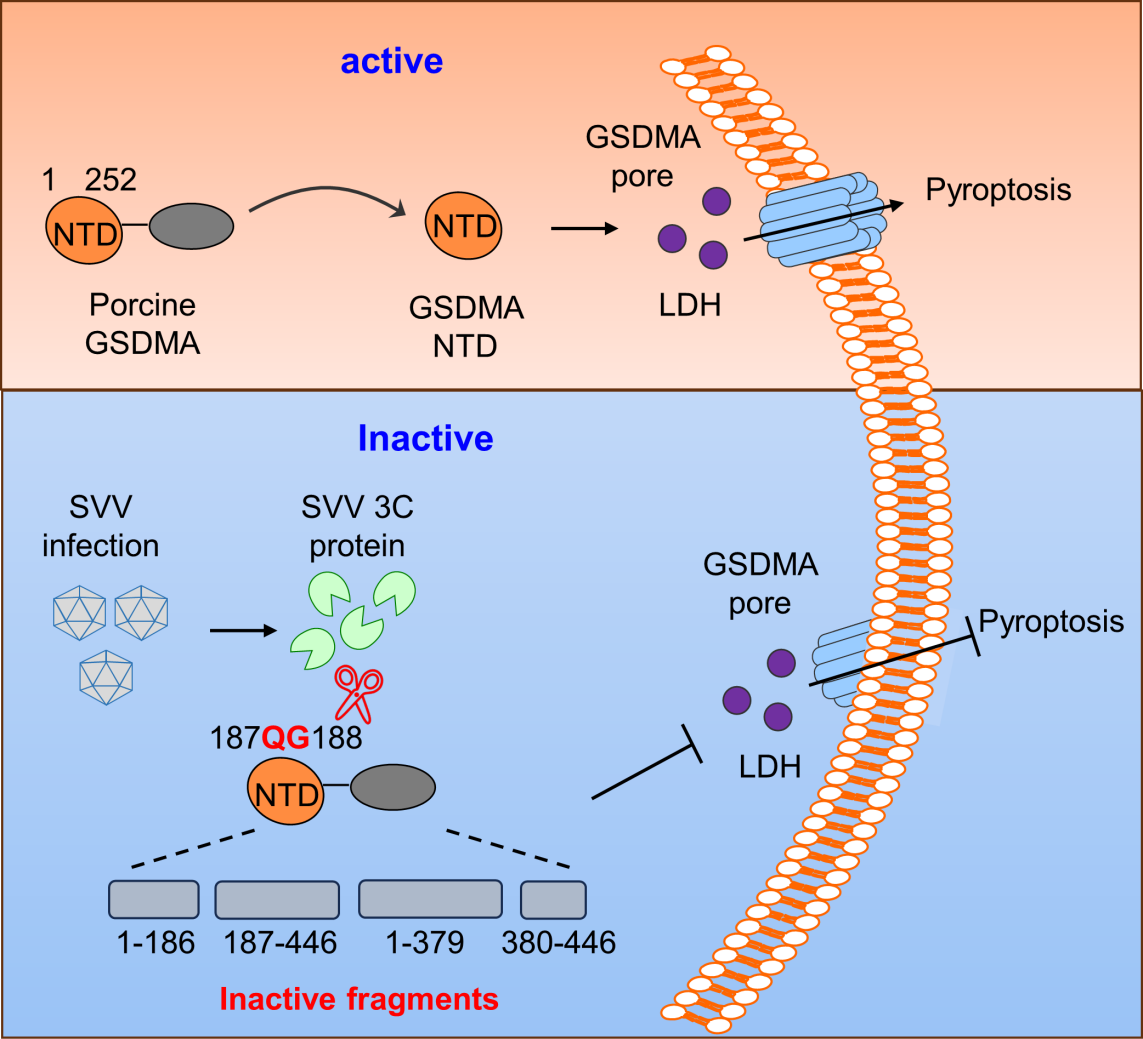
Mechanistic diagram illustrating antagonization of GSDMA-mediated pyroptosis by SVV 3C protease. To evade the innate immune response, SVV evolved a strategy that disrupts pGSDMA NTD-mediated pyroptosis by viral protease 3C at Q187 and G188.

Recent studies have shown that GSDM-mediated pyroptosis plays a crucial role in host defense by regulating viral replication, with GSDMA identified as a novel pyroptosis effector (13, 26–28). To further investigate the effects of pGSDMA_1–186_ on viral replication, pGSDMA_1–186_, pGSDMA_1–252_, and other short fragments were overexpressed in HEK-293T cells, followed by infection with SVV. As expected, we observed that the mRNA levels of SVV were significantly decreased when expressing pGSDMA_1–252_, but not pGSDMA_1–186_ (Fig. 6E). In addition, we also observed that pGSDMA_1–186_ had no effect on the number of GFP^+^ cells after infection with SVV-GFP (Fig. 6F). Similarly, SVV replication did not change when overexpressing hGSDMA_1–185_ compared with hGSDMA_1–251_ transfection group (Fig. S7E and F). Taken together, these results further confirmed that pGSDMA_1–186_ and hGSDMA_1–185_ generated by SVV 3C cleavage do not induce pyroptosis and disrupts the host antiviral innate responses to facilitate SVV replication (Fig. 7). Furthermore, to assess the protease specificity of picornaviruses, HEK-293T cells were co-transfected with pGSDMA and hGSDMA alongside 3C protease from other picornaviruses, including EV71, hepatitis A virus (HAV), CVB3, poliovirus (PV), or encephalomyocarditis virus (EMCV). We did not observe the cleavage band of pGSDMA and hGSDMA compared with SVV 3C protease (Fig. S8). Taken together, these findings confirm that pGSDMA and hGSDMA are specifically cleaved by SVV 3C protease, rather than by 3C protease from other picornaviruses.

## DISCUSSION

GSDMs, a family of pore-forming proteins, are structurally composed of two domains, encompassing members including GSDMA, GSDMB, GSDMC, GSDMD, and GSDME (1, 4). The NTDs of these proteins can insert into and oligomerize within the plasma membrane, triggering lytic cell death and pyroptosis upon removal of the autoinhibitory CTDs. Mirroring the pore-forming activity of hGSDMA_1–251_ (1, 4), our research has established that that pGSDMA_1–252_ can also localize to the plasma membrane and induce cell pyroptosis. In addition, Li et al. have demonstrated that only GSDMA_1–241_ fragment produced by pcaspase-4, but not the intact GSDMA or other cleaved fragments, is capable of inducing typical pyroptosis (28). There are two transcripts for pGSDMA. The GSDMA_1–241_ fragment reported by Li et al. corresponds to pGSDMA X2 (GenBank: XP_013835135.1), aligning with the sequences of pGSDMA_1–252_ in pGSDMA X1 (GenBank: XP_003131545.1) analyzed in our study (Fig. S9A). Consistent with these results, pGSDMA_1–252_ induced pyroptosis as evidenced by changes in morphological features, PI staining and LDH release analysis (Fig. 1). This study reveals that SVV 3C protease can cleave pGSDMA and disrupt its activation upon cleavage by other enzymes, designating pGSDMA as a novel target of SVV 3C. The suppression of SVV replication by pGSDMA_1–252_ underscores the critical role of pGSDMA in the immune defense against viral infection. However, the exact mechanism by which SVV 3C-modulated pGSDMA contributes to viral immune evasion remains to be elucidated.

It is known that pGSDMD-p30, cleaved by porcine caspase-1 at D279, is active and capable to induce pyroptosis (6). Previous experiments have shown that several viral proteases suppress the host antiviral immune responses by disrupting pGSDMD NTD activation. For example, the pGSDMD-N_1–107_ fragment, resulting from cleavage by ASFV protease pS273R at G107, impairs the activity of pGSDMD-N_1–279_, facilitating in ASFV replication (17). Furthermore, coronaviruses proteases, such as PEDV and SARS-CoV-2 Nsp5, cleave pGSDMD at the Q193-G194 junction, generating two fragments incapable of triggering pyroptosis (18). Similarly, EV71 evades the antiviral response by disrupting the function of pGSDMD_1–279_ at Q193-G194 through 3C protease (15). Therefore, it is plausible to extrapolate that pGSDMA_1–186_ fragment, produced by SVV 3C cleavage, could similarly interfere with the lytic ability of pGSDMA_1–252_, thereby inhibiting cell pyroptosis. Consistent with this hypothesis, mutagenesis and functional studies showed that SVV 3C cleaves pGSDMA at the site of Q187-G188, yielding a shorter fragment that suppresses lytic cell death. Notably, RT-PCR and fluorescence analysis further confirmed that pGSDMA_1–186_ fragment has no effect on SVV replication because it cannot cause pyroptosis. In summary, the shorter pGSDMA_1–186_ NT fragment generated by SVV 3C cleavage, is not capable to form pores on the membrane and induce pyroptosis, thereby ensuring viral immune evasion.

An intriguing observation from this study is that SVV 3C protease cleaves pGSDMA into two NT fragments, approximately 25 and 40 kDa. Mass spectrometry pinpointed K379 as the cleavage site of the 40 kDa pGSDMA NT fragment. Surprisingly, we could still detect the cleavage band of pGSDMA NT (~40 kDa), regardless of mutating any residue close to K379. Meanwhile, we further confirmed the 40 kDa pGSDMA NT fragment is the intermediate product for further processing by SVV 3C protease, which yielded an eventual cleavage product (~25 kDa). On the other hand, pGSDMA NT fragment (~40 kDa) could be endogenously cleaved to generate the active NTD, a potential inhibitory fragment. Although we failed to detect the correct cleavage sites of pGSDMD NT fragment (~40 kDa), we confirmed that the cleavage site of pGSDMA NT band (~25 kDa) as Q187-G188.

Previous research has highlighted that cysteine protease SpeB, a virulence factor of group A *Streptococcus*, cleaves human GSDMA at Q246, generating an NT fragment hGSDMA_1–245_ that induces pyroptosis (27). Consistently, another study showed that residues 240–247 in hGSDMA were identified as cleavage motif of SpeB (26). Since these two NT fragments (1–245 and 1–239) are very close to the pore-forming fragment 1–251, they both activate lytic cell death and pyroptosis. Current research has demonstrated that caspase and granzyme cleave the linker regions of and activate GSDMB, GSDMC, GSDMD, and GSDME (5, 22–24). Interestingly, caspase-1 was reported to cleave and activate GSDMA in non-mammals (25). We further found porcine caspase-1 also could not cleave pGSDMA (data not shown). Subsequently, Li et al. have revealed that pcaspase-3 and pcaspase-4 are the endogenous enzymes responsible for activating pGSDMA (28). To determine if similar caspase activation occurs during SVV infection in PAMs, we infected PAMs with SVV and detected the active forms of pcaspase-3 and pcaspase-4. Our immunoblotting results revealed a significant, dose-dependent increase in the active form of pcaspase-3 during SVV infection, whereas the activation of pcaspase-4 was much weaker (Fig. S9B and C), suggesting that SVV-induced pyroptosis is mediated by the cleavage of pGSDMA with pcaspase-3 and pcaspase-4. We also repeated the experiment by co-transfecting V5-pGSDMA with pcaspase-3 or pcaspase-4 into HEK-293T cells. Our observations confirmed the cleavage into an NT fragment (~12 kDa) by pcaspase-3 and an NT fragment (~30 kDa) by pcaspase-4, respectively (Fig. S9D). Notably, SVV infection resulted in the cleavage of pGSDMA into two fragments (~40 and 25 kDa) at the endogenous level, rather than producing the activated fragment (~30 kDa) generated by pcaspase-4. We speculated that SVV 3C protease might obscure the ~30 kDa band by cleaving the activated form pGSDMA X2_1–241_, or that SVV infection only weakly activates pcaspase-4. To verify this, upon co-expression of V5-pGSDMA, Flag-3C, and HA-pcaspase-4 in HEK-293T cells, we observed a weaker band of pGSDMA X2_1–241_ (~30 kDa) compared to the group with co-transfection of pcaspase-4 alone, suggesting that SVV 3C induces an additional cleavage of pGSDMA products (Fig. S9E). This suggests that the SVV 3C-generated pGSDMA_1–186_ fragment competes with the active fragment by pcaspase-4, hindering the formation of the pore-forming pGSDMA X2_1–241_ fragment and facilitating immune evasion. Human GSDMA is mainly expressed in the skin (26, 27), and similarly, the highest expression tissue of pGSDMA is also in the skin. SVV infection causes vesicular skin disease, including vesicles on the snout, oral mucosa, feet, interdigital space, and dewclaws (30), which highlight a key role of pGSDMA in the skin in the defense against SVV infection.

In conclusion, these studies reveal a novel mechanism of immune evasion whereby SVV 3C protease specifically cleaves the pore-forming domain of GSDMA and disrupts its activity to induce pyroptosis, thus enabling viral replication and pathogenesis.

## MATERIALS AND METHODS

### Cells and virus

HEK-293T cells, PK-15 cells, and Hela cells were cultured in Dulbecco’s Modified Eagle Medium (MACGENE, #CM10013) containing 10% fetal bovine serum (PlantChemMed, #PC-00001) at 37°C with 5% CO_2_, whereas primary PAMs from 1-month-old specific pathogen free (SPF) piglets were maintained in RPMI-1640 medium. The SVV and SVV-GFP virus were stored in our laboratory.

### Antibodies

The following antibodies were used in this study. Rabbit anti-V5 monoclonal antibody (mAb) (#13202S), rabbit anti-caspase-4 pAb (#4450S), rabbit anti-caspase-3 mAb and rabbit anti-cleaved caspase-3 (#Asp175), anti-mouse IgG horseradish peroxidase (HRP)-linked antibody, and anti-rabbit IgG HRP-linked antibody were purchased from Cell Signaling Technology (Beverly, MA). Rabbit anti-GAPDH pAb (#10494-1-AP), coraLite594-conjugated goat anti-mouse IgG(H + L) (#SA00013-3), and coraLite488-conjugated goat anti-rabbit IgG(H + L) (#SA00013-2) were purchased from Proteintech Group (Chicago, IL). Mouse anti-Flag mAb (#F1804) was obtained from Sigma-Aldrich (MO, USA). Mouse anti-SVV 3C pAb and mouse anti-pGSDMA pAb were prepared by our laboratory.

### Plasmids

The N-terminal V5-tagged pGSDMA (full length, 1–186, 187–446, 1–379, 1–252, ∆376–380, ∆374–382, and ∆372–384 amino acids) and hGSDMA (full length, 1–185, 186–445, 1–378, and 1–251 amino acids) were ligated to pcDNA3.1 vector. The N-terminal V5-tagged pGSDMA (full length, 1–186, 187–446, 1–379, and 1–252 amino acids) and hGSDMA (full length, 1–185, 186–445, 1–378, and 1–251 amino acids) were cloned into His6-SUMO-pET28a vector. Mutant plasmids of pGSDMA and hGSDMA were constructed according to the manufacturer’s instructions of Mut Express II Fast Mutagenesis Kit V2 (Vazyme, #C1002). The pcDNA3.1-Flag-SVV 3C, pcDNA3.1-Flag-SVV 3C H48A, pcDNA3.1-Flag-SVV 3C D84A, pcDNA3.1-Flag-SVV 3C C160A, pcDNA3.1-Flag-SVV 3C H48A/C160A, pcDNA3.1-Flag-EMCV 3C, pcDNA3.1-Flag-EV71 3C, pcDNA3.1-Flag-HAV 3C, pcDNA3.1-Flag-CVB3 3C, and pcDNA3.1-Flag-PV 3C plasmids were stored in our laboratory. The recombinant protein SVV 3C (wild-type, H48A, D84A, C160A, and H48A/C160A) were stored in our laboratory.

### Immunoblotting

HEK-293T cells were transfected with indicated plasmids using Lipofectamine 2000 (Invitrogen) for 24 h post transfection; cells were lysed on ice with IP lysis buffer (Beyotime, #P0013) for 30 min on ice. Extracts were immunoprecipitated with 1 µg of indicated antibodies and protein A/G PLUS-Agarose beads (Santa Cruz Biotechnology, #sc-2003). Whole-cell lysates or immunoprecipitated extracts were then separated by 10% SDS-PAGE gels (EpiZyme, #PG112) and transferred onto polyvinylidene fluoride (PVDF) membrane (Millipore) for immunoblotting with specific antibodies.

### Protein expression and purification

The pGSDMA protein, pGSDMA-QG187AA mutant protein, and SVV SUMO-3C protein were obtained as previously described (31). *E. coli* BL21 (DE3) cells harboring His6-SUMO-pET28a-pGSDMA, His6-SUMO-pET28a-pGSDMA-QG187AA, and His6-SUMO-pET28a-SVV 3C plasmids were grown in LB medium with 50 μg/mL kanamycin. When OD at 600 nm (OD_600_) reached 1.0, the proteins were induced overnight at 16°C with 0.01 mM IPTG (#18070). The bacteria were lysed in buffer containing 0.05 M Tris-HCl (pH 8.0), 0.3 M NaCl. The proteins were then purified by Ni-NTA beads followed by washing with the buffer containing 0.02 M Tris-HCl (pH 7.5), 0.5 M NaCl, and 0.025 M imidazole, and eluting with the buffer containing 0.02 M Tris-HCl (pH 7.5), 0.15 M NaCl, and 250 mM imidazole. The His6-SUMO tag was removed by SUMO protease overnight at 4°C. Then, the proteins were further purified by Hiload 16/600 Superdex 200 pg gel-filtration chromatography with running buffer containing 20 mM Tris-HCl (pH 7.5), 150 mM NaCl, and then purified by resource S ion exchange with buffer A containing 10 mM Tris-HCl (pH 8.0), 100 mM NaCl, and buffer B containing 10 mM Tris-HCl (pH 8.0), 1 M NaCl. The purified proteins were concentrated to ~12 mg/mL and frozen in liquid nitrogen immediately. All purified proteins were stored in running buffer with 5 mM dithiothreitol (DTT).

Recombinant SUMO-pGSDMA-p40 (1–379 aa) protein was refolded by 8 M urea and dialyzed against multiple changes of phosphate-buffered saline.

### Immunofluorescence and confocal immunofluorescence assay

Hela cells and PK-15 cells were seeded into 24-well plates containing slides. When confluence reached up to 60%–70%, the cells were transfected with the indicated plasmids, and then were washed with phosphate buffer saline (PBS) and fixed for 30 min in 4% paraformaldehyde (Solarbio, #P1110). Cells were then permeabilized for 5 min with 0.1% Triton X-100 and then blocked with 5% bovine serum albumin (Sigma-Aldrich, #A7906-100G) for 0.5 h. Cells were incubated with the appropriate primary antibodies for 1 h and then stained with Alexa Fluor 488- or 594-conjugated secondary antibodies for 1 h. Images were acquired using a laser scanning confocal microscope with Nikon A1 confocal microscope. Images were collected and analyzed by NIS-Elements AR.

### Microscopy

HEK-293T cells were seeded into 24-well plates containing slides. When confluence reached up to 60%–70%, the cells were transfected with the indicated plasmids. Static bright-field cell images were analyzed using Falcon S400, intelligent cell imaging, and analysis system (Alicelligent Technologies).

### Cell cytotoxicity

Cell death was analyzed by using a CytoTox 96 nonradioactive cytotoxicity assay kit (Promega, #G1780) according to LDH released into medium.

### Propidium iodide assay

HEK-293T cells were seeded into 24-well plates and transfected with indicated plasmids. After 24 h, the cells were stained with propidium iodide (Solarbio, #P8080) at 37°C with 5% CO_2_ for 30 min and then analyzed with Evos FL Auto2 fluorescence microscope.

### Bactericidal assay

*E. coli* BL21 (DE3) was transformed with pET-28a expressing indicated plasmid. The transformants were cultured in LB medium containing 50 μg/mL kanamycin as above to OD_600_ 0.6. The transformed cells were serially diluted and plated onto LB agar plates containing 50 μg/mL kanamycin with or without 40 mM IPTG. After incubation at 37°C overnight, the colony-forming unit on the plates was counted and statistically calculated.

### *In vitro* cleavage assay and mass spectrometry

For the cleavage assay *in vitro*, 12 µg full-length pGSDMA, pGSDMA-QG187AA mutant, or SUMO-pGSDMA-p40 recombinant protein was incubated with different amounts of purified SVV 3C or SUMO-3C protein (0.1, 1, 5, 10, and 15 µg) in a 25 µL reaction containing 50 mM HEPES (PH 7.5), 3 mM EDTA, 150 mM NaCl, 0.005% (vol/vol) Tween-20, and 10 mM DTT at 37°C for 2 h. The cleavage reaction was terminated by adding SDS-PAGE loading dye. The reaction mixtures were analyzed by SDS-PAGE and stained with Coomassie blue dye for 10 min. The major cleavage product band with ~40 kDa was excised form the SDS-PAGE gel and digested by trypsin protease for mass spectrometry. The mass spectrometry results were analyzed by Matrix Science.

### Reverse transcription and quantitative real-time PCR (qPCR)

Total RNA was extracted by a simple total RNA kit (TIANGEN, #DP419) and was reversely transcribed to cDNA using HiScript II Q RT SuperMix (Vazyme, #R223-01) according to the manufacturer’s protocol. qPCR was performed in triplicated determinants with 2×Taq Pro Universal SYBR qPCR Master Mix (Vazyme, #Q712-02) on a LightCycler 480 II system (Roche, Switzerland). Relative gene expression levels were determined based on the cycle threshold (ΔΔCT) method and normalized to glyceraldehyde-3-phosphate dehydrogenase (GAPDH) expression. The sequences of qPCR primer sequences were as follows: hGAPDH-F: CTCTGCTCCTCCTGTTCGAC, hGAPDH-R: AATCCGTTGACTCCGACCTT; pGAPDH-F: ACATGGCCTCCAAGGAGTAAGA, pGAPDH-R: GACGCCTGCTTCACCACCTTCT; SVV-VP1-F: AACCGGCTGTGTTTGCTAGAG, SVV-VP1-R: GAACTCGCAGACCACACCAA; SVV-3C-F: GAGCCTTTCCAGACGGTTCA, SVV-3C-R: CGTAACTAGCCGAAACGCCA.

### Sequence alignment

We collected amino acid sequences of pGSDMA (GenBank: XM_003131497.3) and other GSDMA homologs from hGSDMA (GenBank: XM_006721832.4), mouse GSDMA_1 (GenBank: XM_006533850.1), and rhesus monkey GSDMA (GenBank: XM_015119551.2). Clustal OMEGAonline software (https://www.ebi.ac.uk/Tools/msa/clustalo/) was used to perform the multiple-sequence alignment.

### Statistical data analysis

All the graphs and relevant statistical tests used in this work were created by GraphPad software (v.9.0). Data were expressed as mean ± SD and statistically analyzed with a two-tailed unpaired Student’s *t*-test. The *P*-values of ≤0.05 were considered significant: *P < 0.05, **P < 0.01, ***P < 0.001, ****P < 0.0001; ns, no significance.

## SUPPLEMENTAL MATERIAL

Fig. S1 mbio.01680-24-s0001.tif Cross-species amino acid sequence alignment of GSDMA.

Fig. S2 mbio.01680-24-s0002.tif Production of polyclonal antibody specific to pGSDMA.

Fig. S3 mbio.01680-24-s0003.tif Recombinant SVV 3C protease cleaves pGSDMA protein at ~40 kDa, but no mutants can rescue the cleavage.

Fig. S4 mbio.01680-24-s0004.tif Recombinant full-length or truncated pGSDMA-p40 proteins were cleaved by SUMO-3C.

Fig. S5 mbio.01680-24-s0005.tif Purification of recombinant pGSDMA-QG187AA mutant protein and the requirement of SVV 3C protease activity for pGSDMA cleavage.

Fig. S6 mbio.01680-24-s0006.tif hGSDMA1-185 fails to induce pyroptosis.

Fig. S7 mbio.01680-24-s0007.tif hGSDMA1-185 fails to localize to the membrane and loses its bactericidal activity.

Fig. S8 mbio.01680-24-s0008.tif The pGSDMA and hGSDMA are resistant to cleavage by protease 3C from EMCV, EV71, HAV, CVB3, and PV.

Fig. S9 mbio.01680-24-s0009.tif The activated pGSDMA-p30 protein, produced by pcaspase-4, is cleaved by SVV 3C.

Legends mbio.01680-24-s0010.docx Supplemental figure legends.

ASM does not own the copyrights to Supplemental Material that may be linked to, or accessed through, an article. The authors have granted ASM a non-exclusive, world-wide license to publish the Supplemental Material files. Please contact the corresponding author directly for reuse.
