# Supplementary figures and images for: Seneca Valley virus circumvents Gasdermin A-mediated inflammation by targeting the pore-formation domain for cleavage
Source: mBio. 2024 Aug 29;15(10):e01680-24. doi: 10.1128/mbio.01680-24 (PMC11481571; doi:10.1128/mbio.01680-24)

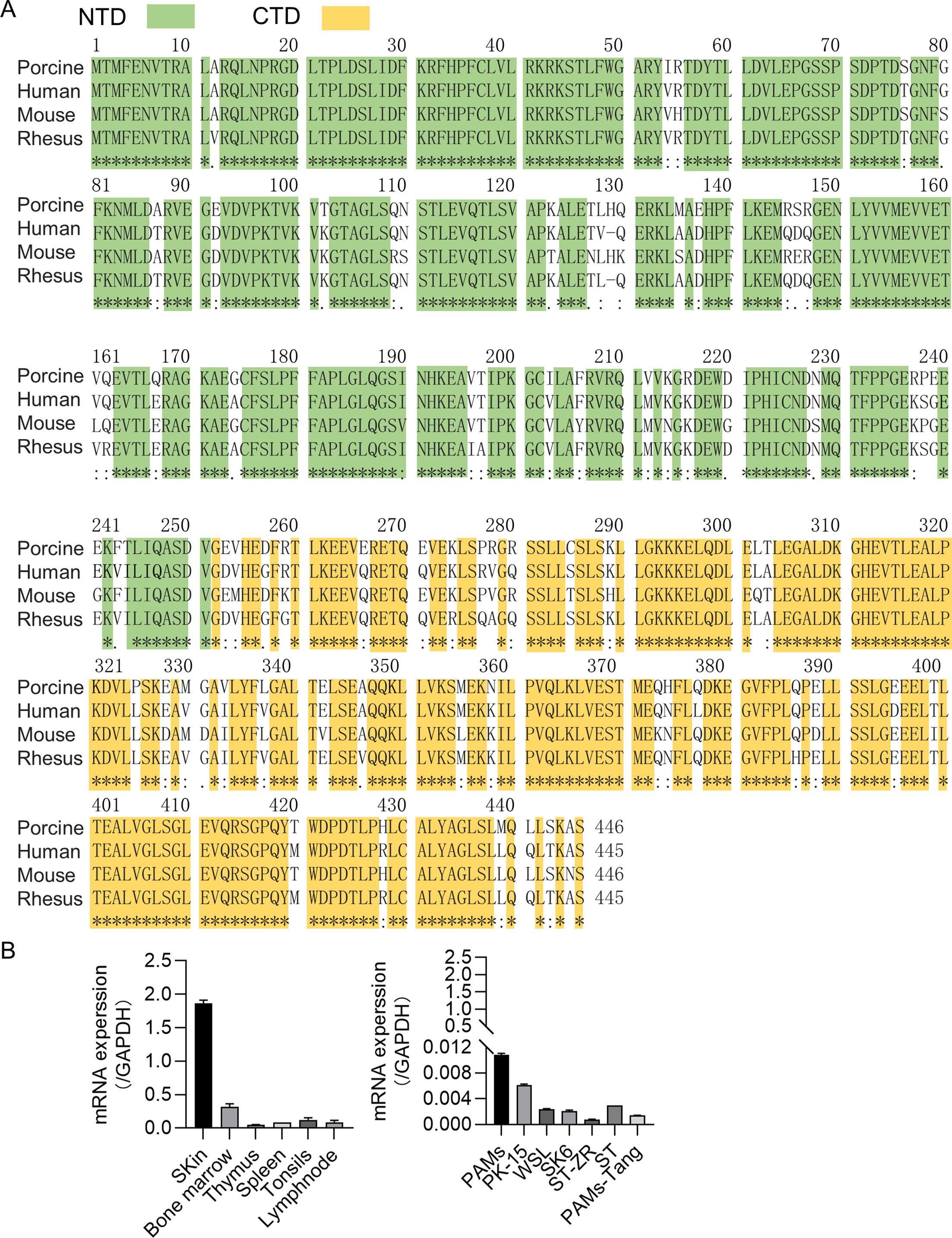

Supplement: Fig. S1 — Cross-species amino acid sequence alignment of GSDMA. [file mbio.01680-24-s0001.tif]

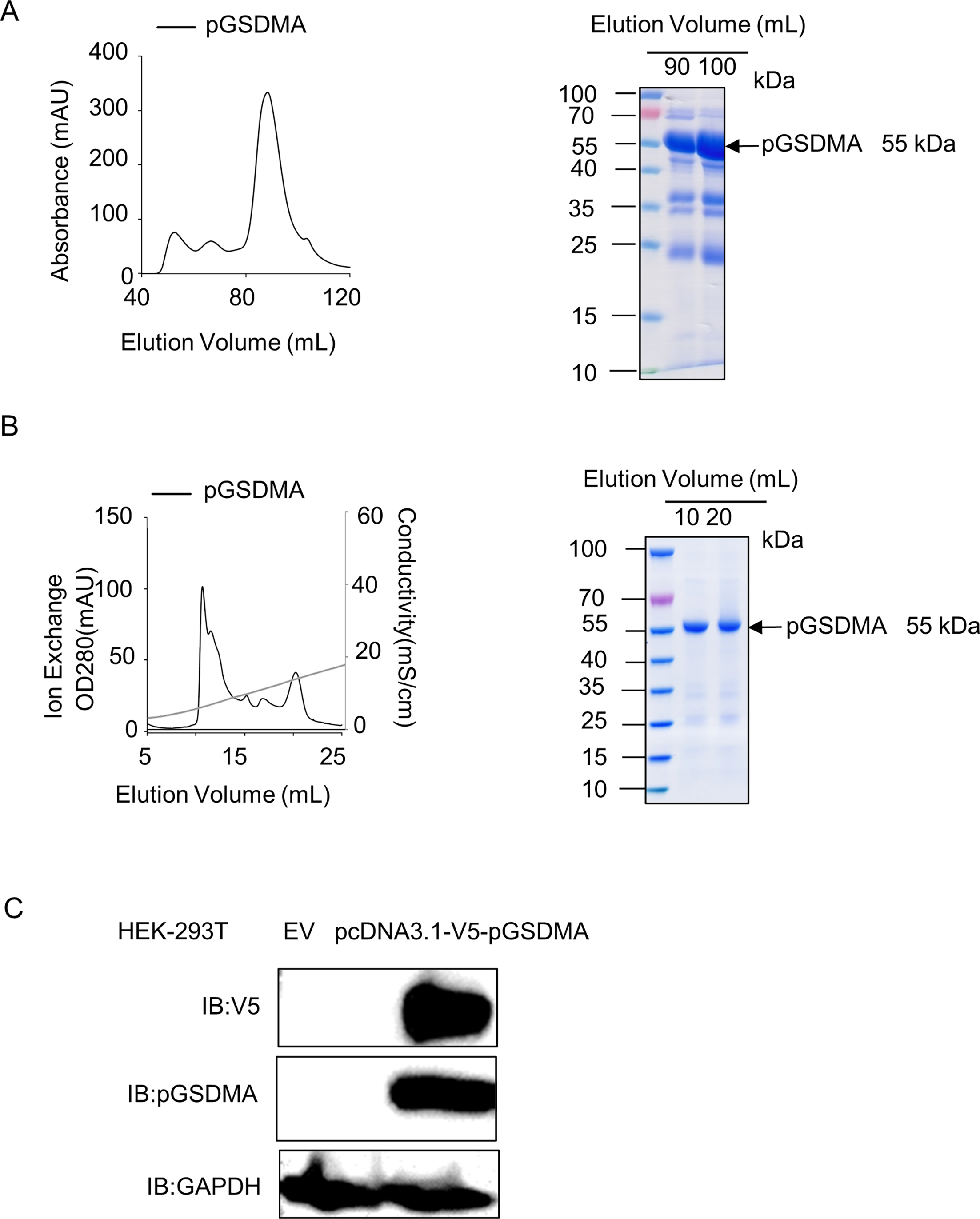

Supplement: Fig. S2 — Production of polyclonal antibody specific to pGSDMA. [file mbio.01680-24-s0002.tif]

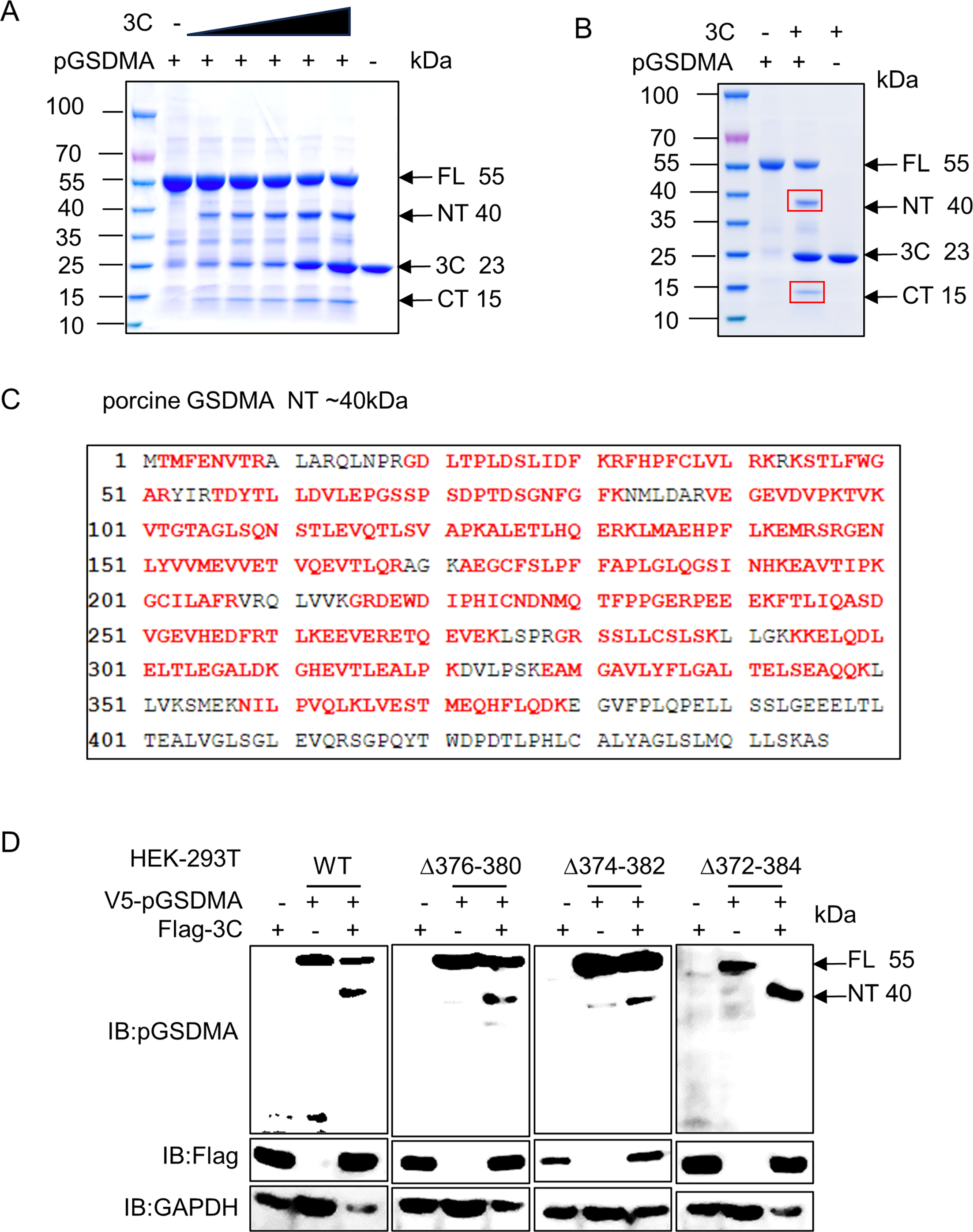

Supplement: Fig. S3 — Recombinant SVV 3C protease cleaves pGSDMA protein at ~40 kDa, but no mutants can rescue the cleavage. [file mbio.01680-24-s0003.tif]

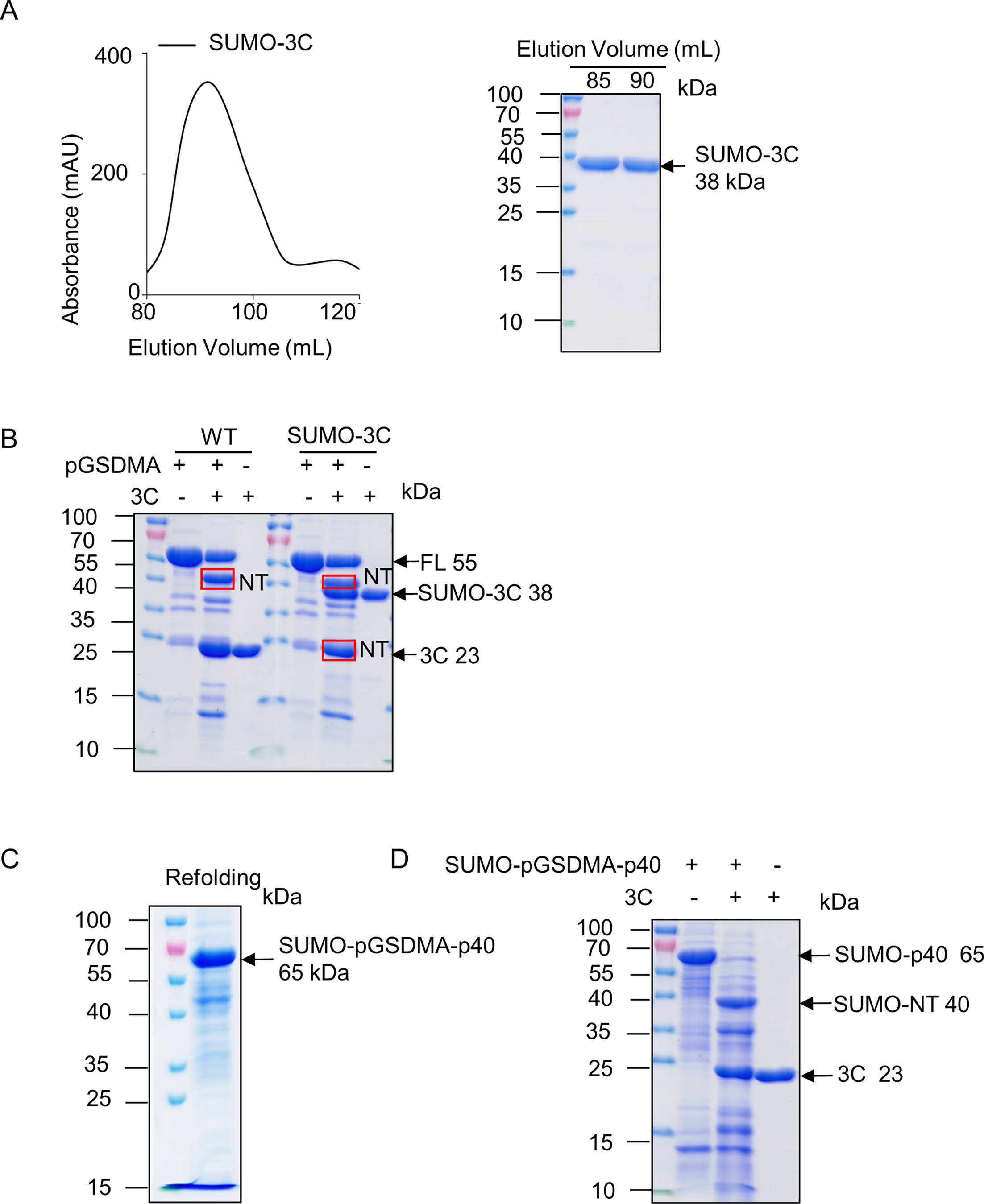

Supplement: Fig. S4 — Recombinant full-length or truncated pGSDMA-p40 proteins were cleaved by SUMO-3C. [file mbio.01680-24-s0004.tif]

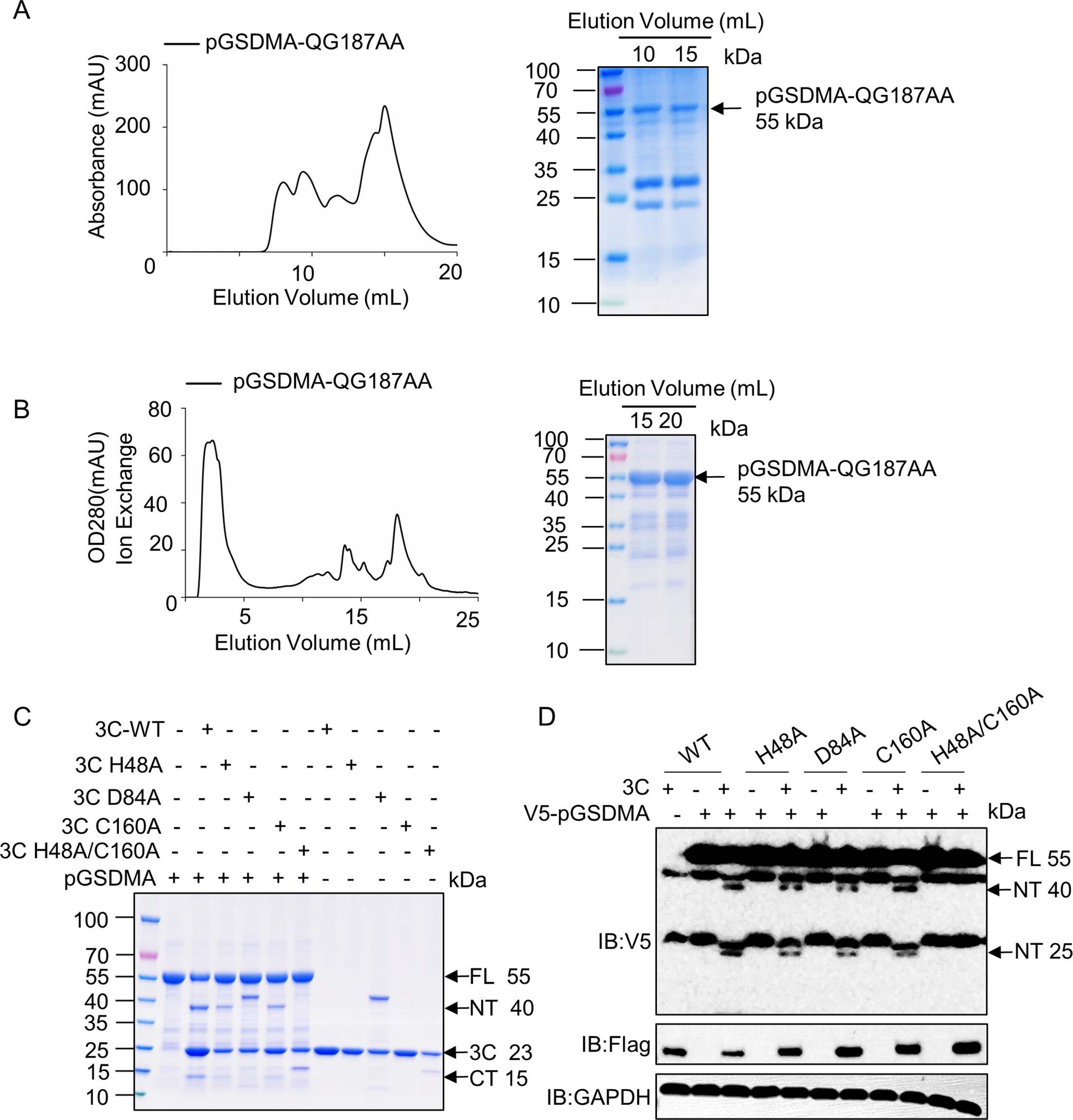

Supplement: Fig. S5 — Purification of recombinant pGSDMA-QG187AA mutant protein and the requirement of SVV 3C protease activity for pGSDMA cleavage. [file mbio.01680-24-s0005.tif]

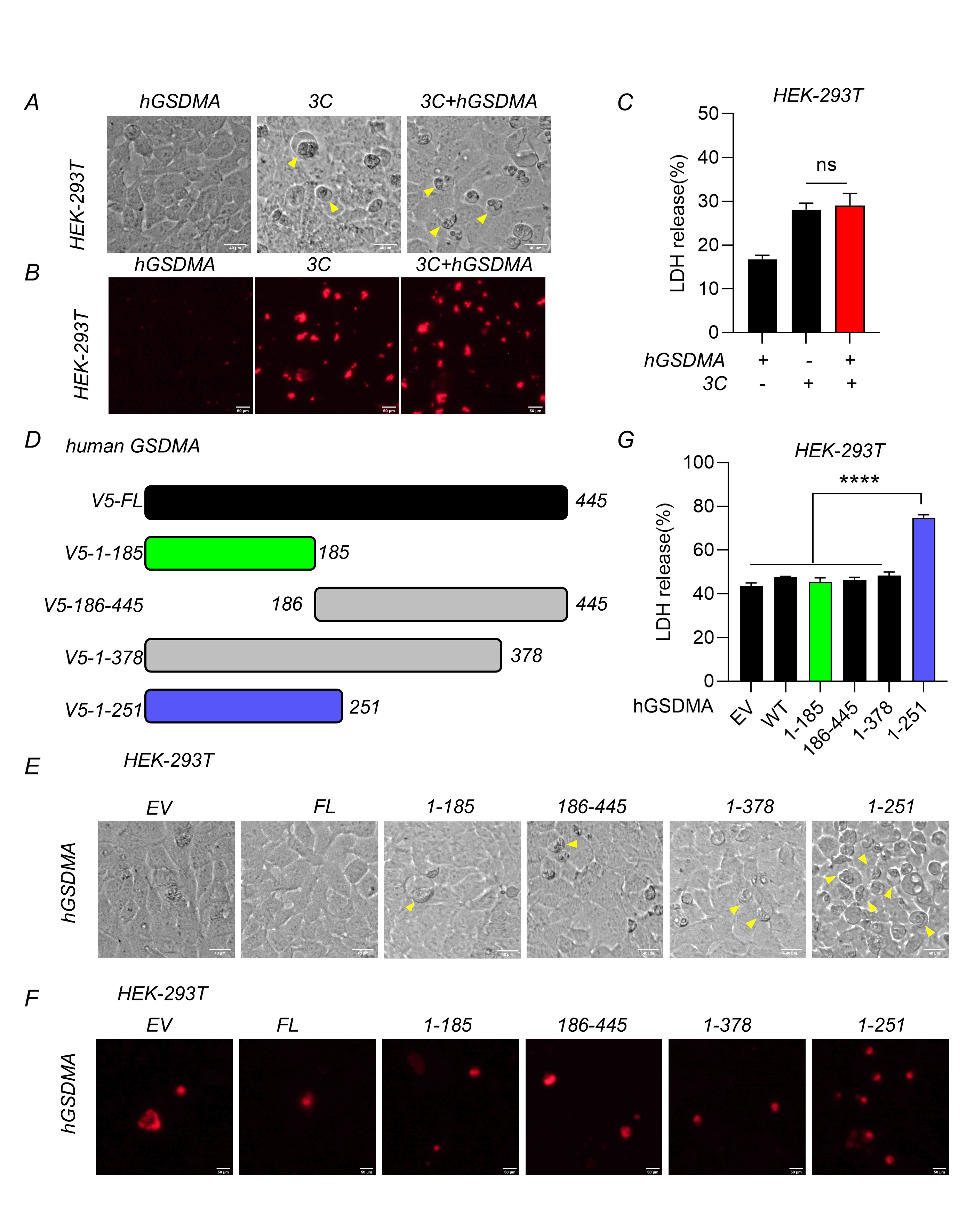

Supplement: Fig. S6 — hGSDMA1-185 fails to induce pyroptosis. [file mbio.01680-24-s0006.tif]

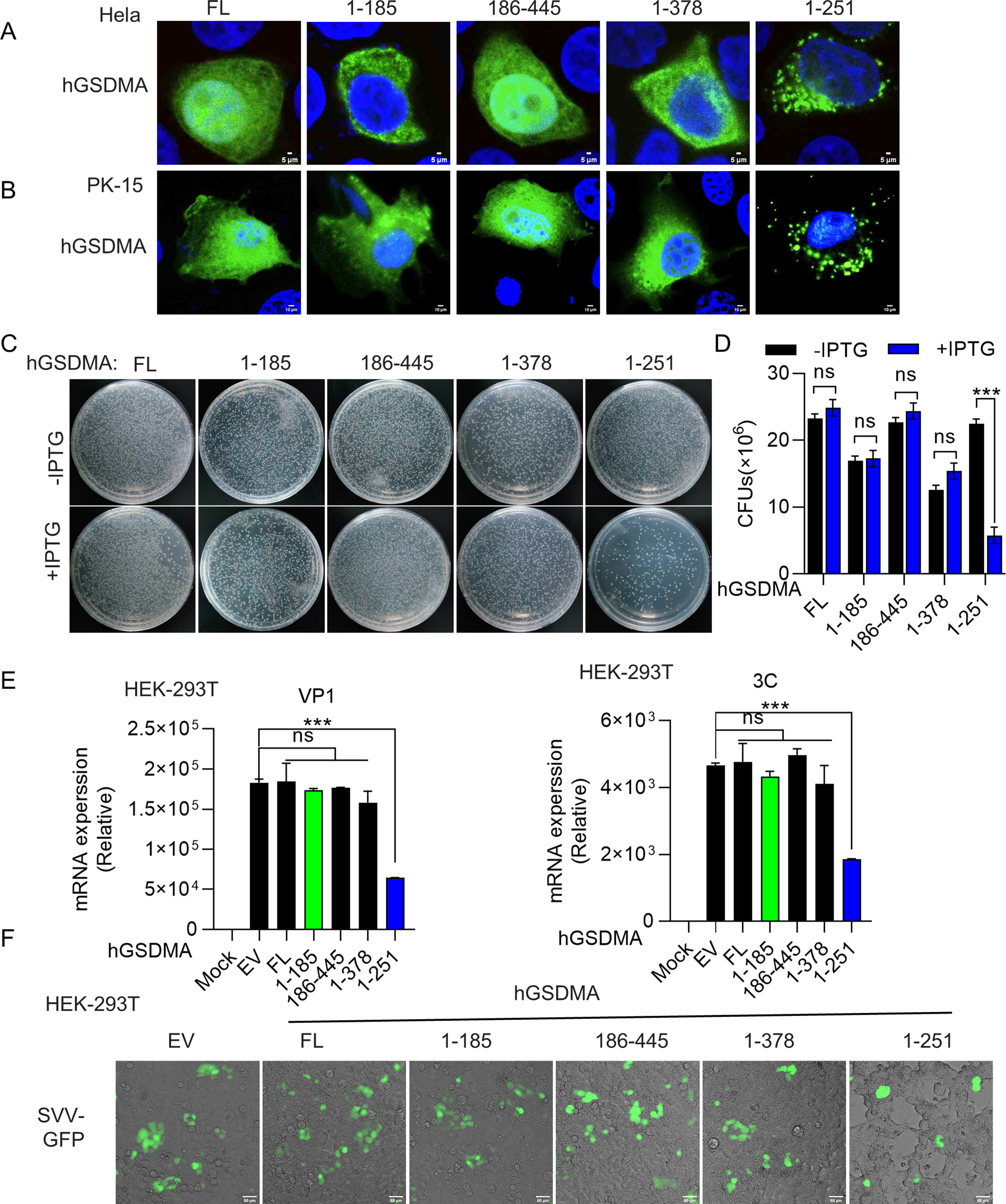

Supplement: Fig. S7 — hGSDMA1-185 fails to localize to the membrane and loses its bactericidal activity. [file mbio.01680-24-s0007.tif]

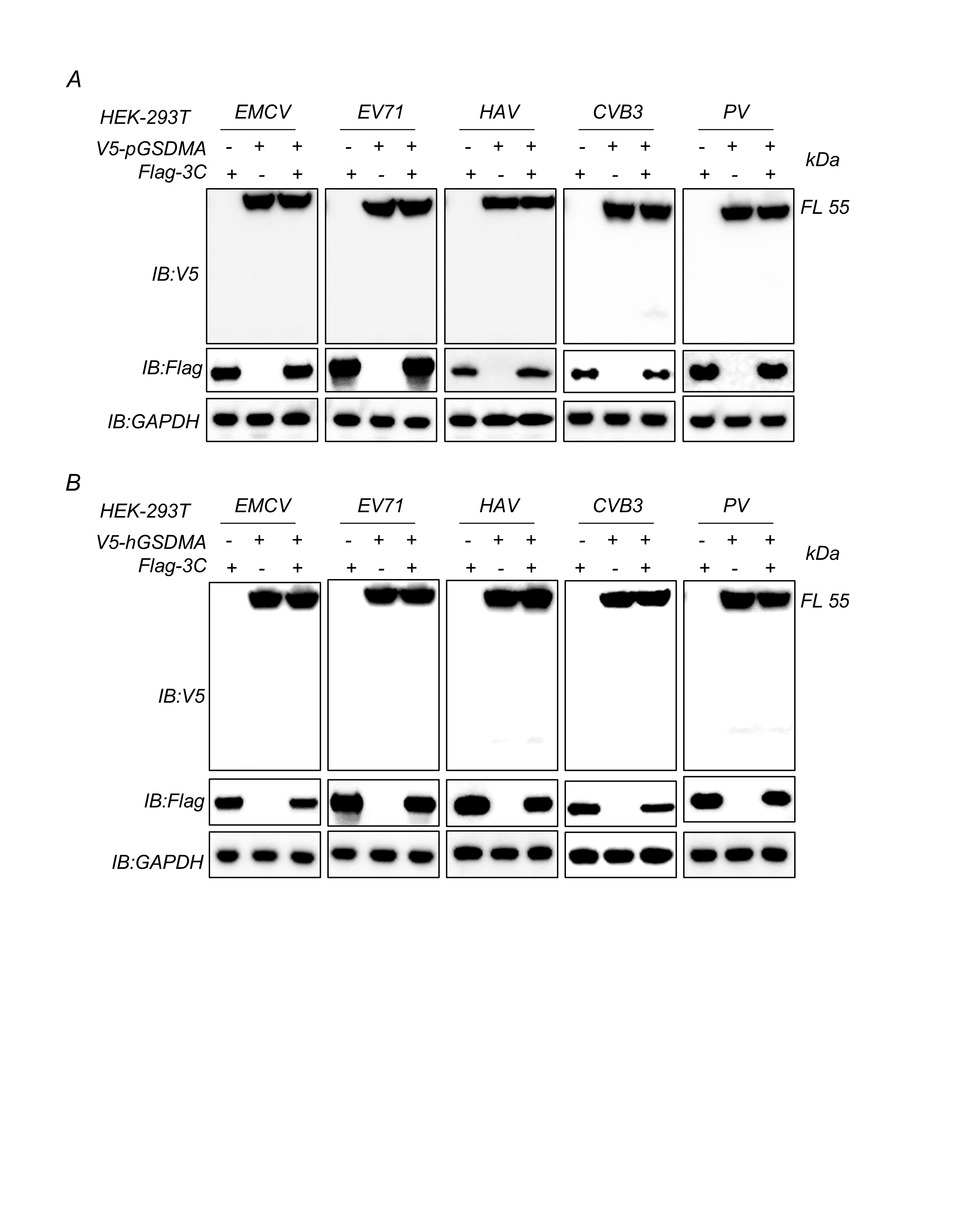

Supplement: Fig. S8 — The pGSDMA and hGSDMA are resistant to cleavage by protease 3C from EMCV, EV71, HAV, CVB3, and PV. [file mbio.01680-24-s0008.tif]

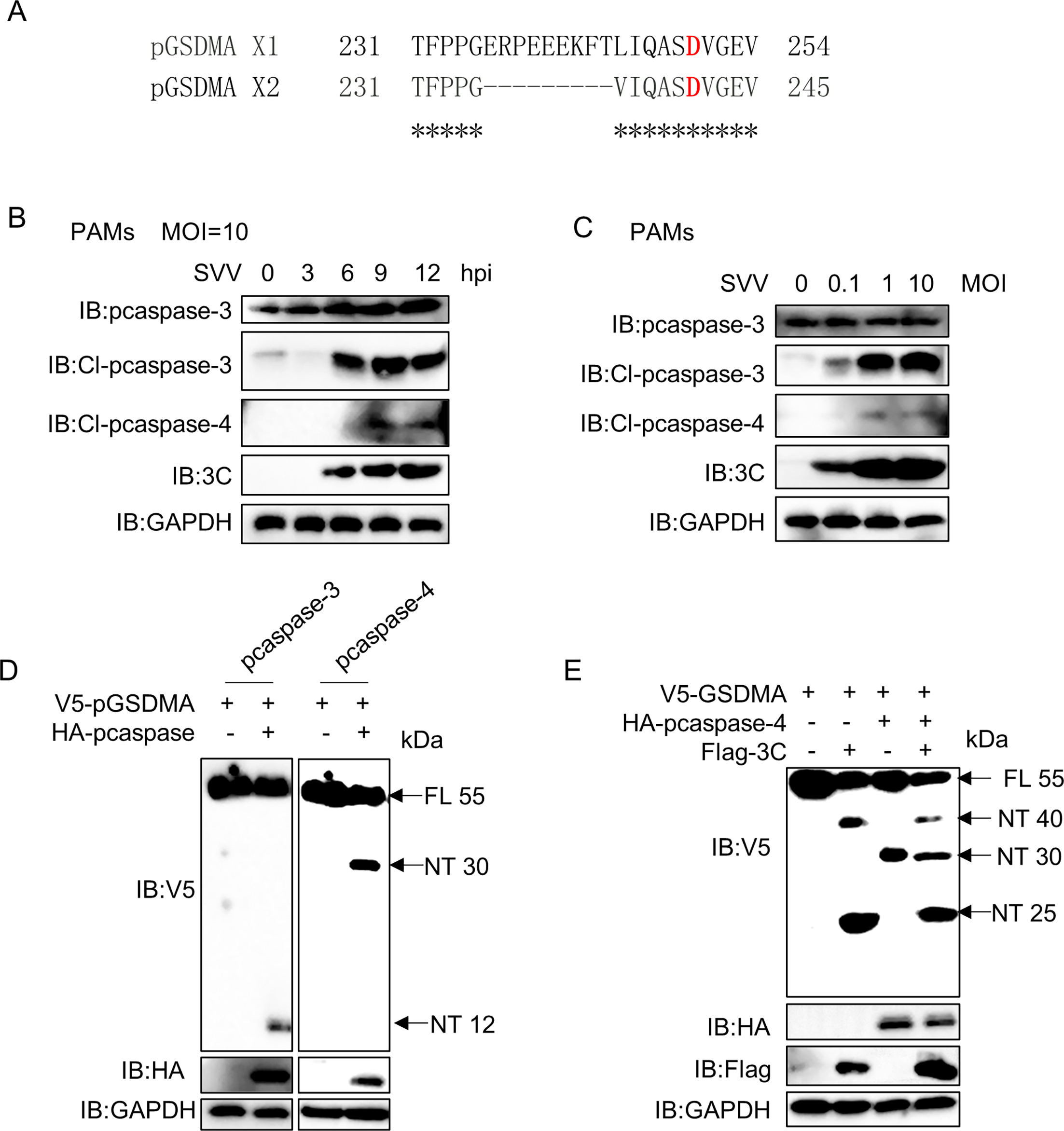

Supplement: Fig. S9 — The activated pGSDMA-p30 protein, produced by pcaspase-4, is cleaved by SVV 3C. [file mbio.01680-24-s0009.tif]
