# Supplementary material for: Seneca Valley virus circumvents Gasdermin A-mediated inflammation by targeting the pore-formation domain for cleavage
Source: mBio. 2024 Aug 29;15(10):e01680-24. doi: 10.1128/mbio.01680-24 (PMC11481571; doi:10.1128/mbio.01680-24)
Supplement: Legends — Supplemental figure legends. [file mbio.01680-24-s0010.docx]

Supplementary Figure legend

Supplementary Figure 1. Cross-species amino acid sequence alignment of GSDMA.

(A) Porcine GSDMA (pGSDMA, Genbank: XM_003131497.3), human GSDMA (hGADMA, Genbank: XM_006721832.4), mouse GSDMA_1 (Genbank: XM_006533850.1) and rhesus monkey GSDMA (Genbank: XM_015119551.2) were aligned by Clustal Omega algorithm. (B) RT-PCR analysis of the mRNA expression of porcine GSDMA in various tissues and cells.

Supplementary Figure 2. Production of polyclonal antibody specific to pGSDMA.

(A and B) SDS-PAGE analysis of full-length recombinant protein pGSDMA purified by gel-filtration chromatography (A) and ion exchange (B). (C) Immunoblotting analysis of the effectiveness of polyclonal antibody directed against pGSDMA in HEK-293T cells mock transfected or transfected with a plasmid encoding full-length pGSDMA for 24 h.

Supplementary Figure 3. Recombinant SVV 3C protease cleaves pGSDMA protein at ~40 kDa but no mutants can rescue the cleavage.

(A) SDS-PAGE analysis of the pGSDMA cleavage in *vitro* in a 25-μL reaction containing 12 μg full-length pGSDMA recombinant protein with different does of SVV 3C recombinant protein (0.1, 0.5, 1, 5, 10 μg) at 37 °C for the indicated time. (B) SDS-PAGE analysis of the pGSDMA cleavage in *vitro* in a 25-μL reaction containing 12 μg full-length pGSDMA recombinant protein with 16 μg SVV 3C recombinant protein for 60 min at 37 °C. (C) Mass spectrometry analysis of the pGSDMA NT band (~40 kDa) cleaved by SVV 3C in *vitro*. The sequence of pGSDMA NT was shown in red. (D) Immunoblotting analysis of pGSDMA NT cleavage band (~40 kDa) in HEK-293T cells co-transfected with plasmids encoding SVV 3C and pGSDMA mutants (WT, ∆376-380, ∆374-382 or ∆372-384) for 24 h.

Supplementary Figure 4. Recombinant full-length or truncated pGSDMA-p40 proteins were cleaved by SUMO-3C.

(A) Purification of recombinant protein SVV SUMO-3C by gel-filtration chromatography. (B) Cleavage of full-length pGSDMA recombinant protein by SVV SUMO-3C. (C) Refolding of SUMO-pGSDMA-p40 (1-379 aa) protein in *vitro*. (D) Cleavage of SUMO-pGSDMA-p40 recombinant protein by purified SVV 3C or SUMO-3C in *vitro*.

Supplementary Figure 5. Purification of recombinant pGSDMA-QG187AA mutant protein and the requirement of SVV 3C protease activity for pGSDMA cleavage.

(A and B) SDS-PAGE analysis of recombinant pGSDMA-QG187AA mutant protein by gel-filtration chromatography (A) and ion exchange (B). (C) SDS-PAGE analysis of pGSDMA cleavage in *vitro* in a 25-μL reaction containing 12 μg full-length pGSDMA recombinant protein with 16 μg SVV 3C (WT, H48A, H84A C160A or H48A/C160A) purified recombinant protein for 60 min at 37 °C. (D) Immunoblotting analysis of pGSDMA cleavage in HEK-293T cells co-transfected with plasmids encoding full-length pGSDMA and SVV 3C mutants (WT, H48A, D84A, C160A or H48A/C160A) for 24 h.

Supplementary Figure 6. hGSDMA_1–185_ fails to induce pyroptosis.

(A-C) HEK-293T cells were transfected with plasmids encoding SVV 3C and full-length hGSDMA for 24 h. (A) Morphological changes characteristics of pyroptosis were visualized using light microscopy, with arrows highlighting pyroptotic cells. Scale bar, 40 μm. (B) The cells were stained with PI for 30 min at 37 °C and then analyzed with fluorescence microscopy. Scale bar, 50 μm. (C) The supernatants were collected and analyzed for LDH levels at OD=490 nm. (D) Schematic representation of V5-hGSDMA and its truncations. (E-G) HEK-293T cells were transfected with a plasmid encoding different truncations (full length, 1-185, 186-445,1-378 or 1-251 aa) hGSDMA for 24 h. (E) Morphological changes characteristics of pyroptosis were visualized using light microscopy, with arrows highlighting pyroptotic cells. Scale bar, 40 μm. (F) The cells were stained with PI and analyzed with fluorescence microscopy. Scale bar, 50 μm. (G) The supernatants were collected and analyzed for LDH levels at OD=490 nm. Data are represented as means ± SD from three biological replicates. ns, no significance, ****p < 0.0001, Student t test.

Supplementary Figure 7. hGSDMA_1-185_ fails to localize to the membrane and loses its bactericidal activity.

(A and B) Immunofluorescence analysis of the localization of hGSDMA cleaved fragments in Hela (A) and PK-15 (B) cells transfected with a plasmid encoding different truncations (full length, 1-185, 186-445,1-378 or 1-251 aa) of hGSDMA for 24 h, followed by fixation and subsequently staining with rabbit monoclonal-specific Abs for V5 (green). Nuclei were stained with DAPI (blue). The fluorescent signals were observed with confocal immunofluorescence microscopy. (A) Scale bar, 5 μm. (B) Scale bar, 10 μm. (C and D) E. coli BL21 was transfected with a pET-28a plasmid encoding different truncations (full length, 1-185, 186-445,1-378 or 1-251 aa) of hGSDMA. The transformants were cultured in LB medium containing 50 μg/ml kanamycin as above to OD600 0.6. Cells were diluted and grown on kan+ LB agar plates with or without 0.4 mM IPTG. After incubation at 37 °C overnight, CFU on the plates was counted and statistically calculated. (D) Cumulative data was shown in the right. (E) RT-PCR analysis of VP1 and 3C mRNA expression of SVV in HEK-293T cells transfected with a plasmid encoding different truncations (full length, 1-185, 186-445,1-378 or 1-251 aa) of hGSDMA for 24 h, followed by infection with SVV (MOI=1) for 24 h. (F) Fluorescence analysis of GFP positive cells in HEK-293T cells processed as above, followed by infection with SVV-GFP (MOI=1). Scale bar, 50 μm. Data are represented as means ± SD from three biological replicates. ns, no significance, ***p < 0.001, Student t test.

Supplementary Figure 8. The pGSDMA and hGSDMA are resistant to cleavage by protease 3C from EMCV, EV71, HAV, CVB3 and PV.

(A and B) Immunoblotting analysis of pGSDMA (A) or hGSDMA(B) cleavage in HEK-293T cells co-transfected with plasmids encoding pGSDMA or hGSDMA and 3C proteases from EMCV, EV71, HAV, CVB3 or PV for 24 h.

Supplementary Figure 9. The activated pGSDMA-p30 protein, produced by pcaspase-4, is cleaved by SVV 3C.

(A) The alignment of amino acid sequence (231-254 aa) between pGSDMA X1 (Genbank: XP_003131545.1) and pGSDMA X2 (Genbank: XP_013835135.1) by the Clustal Omega algorithm. Red, the cleavage site of pcaspase-4. (B and C) Immunoblotting analysis of the protein expression levels of pcaspase-3, cleaved-pcaspase-3, cleaved-pcaspase-4 in PAMs infected with SVV across different time points (B) and at various MOI (C). (D) Immunoblotting analysis of the cleavage pGSDMA in HEK-293T cells following co-transfection with plasmids encoding pcaspase-3 or pcaspase-4 along with full-length pGSDMA for 24 h. (E) Immunoblotting analysis of the pGSDMA cleavage in HEK-293T cells following co-transfection with plasmids encoding SVV 3C, pcaspase-4 or both SVV 3C and pcaspase-4 along with full-length pGSDMA for 24 h.
